# Inhaled corticosteroids in COPD and onset of type 2 diabetes and osteoporosis: matched cohort study

**DOI:** 10.1038/s41533-019-0150-x

**Published:** 2019-10-28

**Authors:** David B. Price, Jaco Voorham, Guy Brusselle, Andreas Clemens, Konstantinos Kostikas, Jeffrey W. Stephens, Hye Yun Park, Nicolas Roche, Robert Fogel

**Affiliations:** 1 Observational and Pragmatic Research Institute, Singapore, Singapore; 2 Academic Primary Care, University of Aberdeen, Aberdeen, UK; 3 Ghent University Hospital, Ghent, Belgium, and Erasmus Medical Center Rotterdam, Rotterdam, The Netherlands; 4 Novartis Pharma AG, Basel, Switzerland; 5 Department of Cardiology and Angiology I, Heart Center Freiburg University, Faculty of Medicine, University of Freiburg, Freiburg, Germany; 6 Swansea University Medical School, Swansea University, Swansea, UK; 7 Division of Pulmonary and Critical Care Medicine, Department of Medicine, Samsung Medical Center, Sungkyunkwan University School of Medicine, Seoul, South Korea; 8 Hôpital et Institut Cochin (UMR1016), Assistance Publique Hôpitaux de Paris Centre Université de Paris, Paris, France; 9 Novartis Pharmaceuticals Corporation, East Hanover, NJ USA; 10 Present Address: Respiratory Medicine Department, University of Ioannina, Ioannin, Greece

**Keywords:** Outcomes research, Chronic obstructive pulmonary disease

## Abstract

Some studies suggest an association between onset and/or poor control of type 2 diabetes mellitus and inhaled corticosteroid (ICS) therapy for chronic obstructive pulmonary disease (COPD), and also between increased fracture risk and ICS therapy; however, study results are contradictory and these associations remain tentative and incompletely characterized. This matched cohort study used two large UK databases (1983–2016) to study patients (≥ 40 years old) initiating ICS or long-acting bronchodilator (LABD) for COPD from 1990–2015 in three study cohorts designed to assess the relation between ICS treatment and (1) diabetes onset (*N* = 17,970), (2) diabetes progression (*N* = 804), and (3) osteoporosis onset (*N* = 19,898). Patients had ≥ 1-year baseline and ≥ 2-year outcome data. Matching was via combined direct matching and propensity scores. Conditional proportional hazards regression, adjusting for residual confounding after matching, was used to compare ICS vs. LABD and to model ICS exposures. Median follow-up was 3.7–5.6 years/treatment group. For patients prescribed ICS, compared with LABD, the risk of diabetes onset was significantly increased (adjusted hazard ratio 1.27; 95% CI, 1.07–1.50), with overall no increase in risk of diabetes progression (adjusted hazard ratio 1.04; 0.87–1.25) or osteoporosis onset (adjusted hazard ratio 1.13; 0.93–1.39). However, the risks of diabetes onset, diabetes progression, and osteoporosis onset were all significantly increased, with evident dose–response relationships for all three outcomes, at mean ICS exposures of 500 µg/day or greater (vs. < 250 µg/day, fluticasone propionate–equivalent). Long-term ICS therapy for COPD at mean daily exposure of ≥ 500 µg is associated with an increased risk of diabetes, diabetes progression, and osteoporosis.

## Introduction

The identification of patients who are most likely to benefit from inhaled corticosteroids (ICS) for chronic obstructive pulmonary disease (COPD) remains an important topic of research and of interest for the clinical community.^1–3^ In addition, the adverse effects of ICS therapy for COPD—the risk side of the risk-benefit equation—remain incompletely characterized and require further investigation.^4^

Well-accepted adverse effects associated with ICS therapy in COPD include increased risk of pneumonia, as well as skin bruising, oropharyngeal candidiasis, hoarse voice, and increased risk of tuberculosis.^4–6^ Other potential adverse effects of ICS are less well-characterized and considered not definitive in the current Global Initiative for Chronic Obstructive Lung Disease (GOLD) strategy publication, such as the risks of diabetes, poor control of diabetes, decreased bone density, and fracture.^4^ Although some cohort studies report increased risk of onset and progression of diabetes, particularly at higher ICS doses,^7,8^ other cohort studies and reviews of controlled trial results find no such association.^9,10^ Similarly, discordant results have been published for the association of ICS with risk of fracture,^6,11^ although a recently published large case–control study of patients with COPD followed until March 2007 found that long-term, high-dose ICS was associated with a modest increase in the risk of hip and upper extremity fractures.^12^

Many prior studies of ICS adverse effects for patients with COPD suffer from important limitations. In particular, most randomized controlled trials are not sufficiently powered or long enough to evaluate adverse effects. Moreover, many observational studies have been criticized for patient selection and time-related biases,^3^ and patients with concomitant asthma may not be specifically excluded.^3,7,8^

The current GOLD strategy recommends limiting ICS-containing therapy to patients in GOLD group D who experience frequent COPD exacerbations and persistent symptoms despite optimal bronchodilator therapy with long-acting bronchodilators (LABD) and who have a blood eosinophil count ≥ 300 cells/µL.^4,13^ However, discrepancies have frequently been reported between GOLD recommendations and clinical practice, where ICS are widely prescribed for COPD, typically in combination with long-acting β-agonist (LABA) or as monotherapy,^14–16^ with up to 60% of patients in GOLD groups A and B initiated on ICS-containing therapy in some studies.^16,17^ This is particularly concerning given that patients with COPD are typically older, thus more likely to have pre-existing comorbidities.^2,3,5^

The aim of this large, historical matched cohort study was to evaluate whether ICS therapy for patients with COPD is associated with an increased onset or accelerated progression of type 2 diabetes mellitus, or with an increased onset of osteoporosis. We were able to access large medical record databases to evaluate median ICS exposure durations of over 5 years. Our primary objective was to compare the association of onset and progression of diabetes, and onset of osteoporosis, between patients prescribed ICS (with or without LABDs) compared with LABDs and to examine these outcomes according to different levels of mean daily and cumulative ICS exposure, both for all matched patients and stratified by GOLD groups assigned using the GOLD 2011 strategy.^18^

## Results

### Patients

We identified 152,516 patients in the Clinical Practice Research Datalink (CPRD) and Optimum Patient Care Research Database with a recorded COPD diagnosis preceding maintenance treatment initiation for COPD from 1990 to August 2015, including 104,519 and 47,997 patients initiating ICS and LABD therapy, respectively. Of these, 28,060 (27%) and 9862 (21%) patients, respectively, were eligible for the study after we applied general eligibility criteria and eliminated duplicate patients (see online Supplementary Fig. 1).

Of the total of 37,922 eligible patients, 33,934 (89%), 1346 (3.5%), and 36,154 (95%) were eligible for the diabetes onset, diabetes progression, and osteoporosis onset cohorts, respectively. After matching, the two treatment groups totaled 17,970 patients in the diabetes onset cohort, 804 patients in the diabetes progression cohort, and 19,898 patients in the osteoporosis onset cohort, representing 53%, 60%, and 55% of the unmatched cohorts, respectively. Baseline demographic and clinical characteristics of the unmatched treatment groups of each study cohort are in the online Supplementary Tables 1–3, and baseline characteristics of the matched treatment groups are summarized in Tables 1–3, with additional baseline characteristics reported in Supplementary Tables 4–6.

**Table 1:** Demographic and clinical characteristics of matched patients in the diabetes onset cohort during the baseline year

	LABD (*n* = 6540)	ICS (*n* = 11,430)	*P* value^a^	SMD (%)^b^	RCC (%)^b^
Male, *n* (%)	3835 (58.6)	6788 (59.4)	0.33	1.5	0.0
Age, mean (SD)	68.0 (9.5)	67.7 (9.4)	0.035	3.9	0.3
Index year, median (IQR)	2008 (2006–2011)	2007 (2004–2009)	<0.0001	37.3	1.6
Smoking status, available data, *n* (%)	6479 (99.1)	11,303 (98.9)			
Current smoker	2904 (44.8)	4978 (44.0)	0.33	2.3	0.1
Ex-smoker	3269 (50.5)	5823 (51.5)			
Never-smoker	306 (4.7)	502 (4.4)			
Body mass index, kg/m^2^, mean (SD)	26.5 (5.4)	26.3 (5.3)	0.083	2.8	4.2
Cardiovascular disease, *n* (%)	2233 (34.1)	3619 (31.7)	0.0006	5.3	0.4
OCS prescriptions/yr, *n* (%)^c^					
0	5275 (80.7)	8827 (77.2)	<0.0001	8.3	2.1
1	880 (13.5)	1775 (15.5)			
2	258 (3.9)	530 (4.6)			
≥3	127 (1.9)	298 (2.6)			
Antibiotic prescriptions/yr, *n* (%)^d^					
0	3911 (59.8)	6732 (58.9)	0.151	3.2	0.0
1	1540 (23.5)	2646 (23.1)			
2	660 (10.1)	1219 (10.7)			
≥3	429 (6.6)	833 (7.3)			
FEV_1_ %predicted, data available	4090 (62.5)	5799 (50.7)			
<30%	161 (3.9)	292 (5.0)	<0.0001	9.6	9.2
30–49%	900 (22.0)	1568 (27.0)			
50–79%	2441 (59.7)	3093 (53.3)			
≥80%	588 (14.4)	846 (14.6)			
Exacerbations/yr, *n* (%)^d^					
0	3452 (52.8)	5780 (50.6)	0.0035	5.2	0.0
1	1834 (28.0)	3244 (28.4)			
≥2	1254 (19.2)	2406 (21.0)			
MRC score available, *n* (%)	5895 (90.1)	9615 (84.1)			
1	832 (14.1)	1405 (14.6)	0.25	0.5	8.7
2	2852 (48.4)	4601 (47.9)			
3	1498 (25.4)	2399 (25.0)			
4	623 (10.6)	1019 (10.6)			
5	90 (1.5)	191 (2.0)			
GOLD group data available, *n* (%)	5895 (90.1)	9615 (84.1)			
GOLD A	2476 (42.0)	3807 (39.6)	0.0001	6.6	9.8
GOLD B	1426 (24.2)	2214 (23.0)			
GOLD C	1208 (20.5)	2199 (22.9)			
GOLD D	785 (13.3)	1395 (14.5)			

*FEV*_*1*_ forced expiratory volume in 1 s, *GOLD* Global Initiative for Chronic Obstructive Lung Disease, *ICS* inhaled corticosteroid, *IQR* interquartile range, *LABD* long-acting bronchodilator, *MRC* Medical Research Council dyspnea scale, *OCS* oral corticosteroid, *RCC* relative change in coefficient, *SMD* standardized mean difference, *yr* during the baseline year

For baseline variables with missing data, the percentages of patients with available data are noted

^a^*P* values shown using Kruskal–Wallis equality-of-populations rank test or Pearson’s *χ*^2^-test of independent categories for continuous and categorical variables, respectively

^b^An SMD ≤ 10% indicates sufficient balance between groups. The baseline variables with RCC ≥ 2%, which we defined as indicating bias potential, were selected for the direct matching attempts

^c^38 (0.6%) and 69 (0.6%) patients in LABD and ICS groups were receiving maintenance OCS (see Supplementary Table 4)

^d^Antibiotics were those prescribed on the same day as a lower respiratory consultation (identified by a Read code for a lower respiratory event) Moderate-to-severe exacerbations are defined in Methods section

**Table 2:** Demographic and clinical characteristics of matched patients in the diabetes progression cohort during the baseline year

	LABD (*n* = 324)	ICS (*n* = 480)	*P* value^a^	SMD (%)^b^	RCC (%)^b^
Male, *n* (%)	214 (66.0)	319 (66.5)	0.90	0.9	0.0
Age, mean (SD)	70.8 (8.1)	71.1 (7.5)	0.69	6.1	0.8
Index year, median (IQR)	2009 (2007–2011)	2008 (2006–2010)	0.0009	24.0	1.4
Smoking status, available data, *n* (%)	324 (100.0)	480 (100.0)			
Current smoker	119 (36.7)	154 (32.1)	0.38	10.0	0.0
Ex-smoker	188 (58.0)	301 (62.7)			
Never-smoker	17 (5.2)	25 (5.2)			
Body mass index, kg/m^2^, mean (SD)	30.4 (5.7)	30.8 (6.4)	0.79	7.6	0.0
Last HbA1c, %, mean (SD)	6.9 (1.1)	6.9 (1.1)	0.79	2.5	0.1
Cardiovascular disease, *n* (%)	181 (55.9)	259 (54.0)	0.59	3.8	0.0
OCS prescriptions/yr, *n* (%)^c^					
0	275 (84.9)	388 (80.8)	0.33	13.8	0.1
1	37 (11.4)	63 (13.1)			
2	9 (2.8)	18 (3.8)			
≥ 3	3 (0.9)	11 (2.3)			
Antibiotic prescriptions/yr, *n* (%)^d^					
0	191 (59.0)	276 (57.5)	0.72	8.3	0.8
1	86 (26.5)	120 (25.0)			
2	30 (9.3)	53 (11.0)			
≥ 3	17 (5.2)	31 (6.5)			
FEV_1_ %predicted, data available	224 (69.1)	283 (59.0)			
<30%	7 (3.1)	9 (3.2)	0.69	10.8	15.5
30–49%	49 (21.9)	72 (25.4)			
50–79%	132 (58.9)	165 (58.3)			
≥ 80%	36 (16.1)	37 (13.1)			
Exacerbations/yr, *n* (%)^d^					
0	175 (54.0)	245 (51.0)	0.48	8.8	2.3
1	95 (29.3)	139 (29.0)			
≥ 2	54 (16.7)	96 (20.0)			
MRC score available, *n* (%)	307 (94.8)	459 (95.6)			
1	37 (12.1)	62 (13.5)	0.97	5.5	1.9
2	136 (44.3)	194 (42.3)			
3	86 (28.0)	128 (27.9)			
4	40 (13.0)	63 (13.7)			
5	8 (2.6)	12 (2.6)			
GOLD group data available, *n* (%)	307 (94.8)	459 (95.6)			
GOLD A	118 (38.4)	169 (36.8)	0.83	6.9	1.7
GOLD B	85 (27.7)	120 (26.1)			
GOLD C	55 (17.9)	87 (19.0)			
GOLD D	49 (16.0)	83 (18.1)			

*FEV*_*1*_ forced expiratory volume in 1 s, *GOLD* Global Initiative for Chronic Obstructive Lung Disease, *ICS* inhaled corticosteroid, *IQR* interquartile range, *LABD* long-acting bronchodilator, *MRC* Medical Research Council dyspnea scale, *OCS* oral corticosteroid, *RCC* relative change in coefficient, *SMD* standardized mean difference, *yr* during the baseline year

^a^*P* values shown using Kruskal–Wallis equality-of-populations rank test or Pearson's *χ*^2^-test of independent categories for continuous and categorical variables, respectively

^b^An SMD ≤ 10% indicates sufficient balance between groups. The baseline variables with RCC ≥ 2%, which we defined as indicating bias potential, were selected for the direct matching attempts

^c^1 (0.3%) and 2 (0.4%) patients in LABD and ICS groups were receiving maintenance OCS (see Supplementary Table 5)

^d^Antibiotics were those prescribed on the same day as a lower respiratory consultation (identified by a Read code for a lower respiratory event). Moderate-to-severe exacerbations are defined in Methods section

**Table 3:** Demographic and clinical characteristics of matched patients in the osteoporosis onset cohort during the baseline year

	LABD (*n* = 7279)	ICS (*n* = 12,619)	*P* value^a^	SMD (%)^b^	RCC (%)^b^
Male, *n* (%)	4517 (62.1)	7893 (62.5)	0.48	1.0	0.0
Age, mean (SD)	67.9 (9.4)	67.7 (9.3)	0.034	3.8	0.0
Index year, median (IQR)	2008 (2006–2011)	2007 (2004–2009)	<0.0001	38.1	1.3
Smoking status, available data, *n* (%)	7214 (99.1)	*12,484 (98.9)*			
Current smoker	3161 (43.8)	5394 (43.2)	0.47	1.8	0.1
Ex smoker	3717 (51.5)	6537 (52.4)			
Never smoker	336 (4.7)	553 (4.4)			
Body mass index, kg/m^2^, mean (SD)	27.1 (5.6)	27.1 (5.6)	0.007	3.0	0.4
Cardiovascular disease, *n* (%)	2663 (36.6)	4343 (34.4)	0.0020	4.6	0.4
OCS prescriptions/yr, *n* (%)^c^					
0	5880 (80.8)	9768 (77.4)	<0.0001	8.4	0.6
1	977 (13.4)	1950 (15.5)			
2	286 (3.9)	570 (4.5)			
≥ 3	136 (1.9)	331 (2.6)			
Antibiotic prescriptions/yr, *n* (%)^d^					
0	4375 (60.1)	7440 (59.0)	0.034	3.9	0.0
1	1709 (23.5)	2911 (23.1)			
2	726 (10.0)	1340 (10.6)			
≥ 3	469 (6.4)	928 (7.4)			
FEV_1_ %predicted, data available	4544 (62.4)	*6431 (51.0)*			
< 30%	167 (3.7)	324 (5.0)	<0.0001	10.2	7.6
30–49%	992 (21.8)	1704 (26.5)			
50–79%	2741 (60.3)	3491 (54.3)			
≥ 80%	644 (14.2)	912 (14.2)			
Exacerbations/yr, *n* (%)^d^					
0	3867 (53.1)	6392 (50.7)	0.0011	5.4	0.0
1	2019 (27.7)	3587 (28.4)			
≥ 2	1393 (19.1)	2640 (20.9)			
MRC score available, *n* (%)	6567 (90.2)	*10,635 (84.3)*			
1	912 (13.9)	1548 (14.6)	0.108	0.2	8.1
2	3135 (47.7)	5070 (47.7)			
3	1731 (26.4)	2647 (24.9)			
4	678 (10.3)	1164 (10.9)			
5	111 (1.7)	206 (1.9)			
GOLD group data available, *n* (%)	6567 (90.2)	*10,635 (84.3)*			
GOLD A	2741 (41.7)	4203 (39.5)	<0.0001	5.9	7.9
GOLD B	1623 (24.7)	2490 (23.4)			
GOLD C	1306 (19.9)	2415 (22.7)			
GOLD D	897 (13.7)	1527 (14.4)			

*FEV*_*1*_ forced expiratory volume in 1 s, *GOLD* Global Initiative for Chronic Obstructive Lung Disease, *ICS* inhaled corticosteroid, *IQR* interquartile range, *LABD* long-acting bronchodilator, *MRC* Medical Research Council dyspnea scale, *OCS* oral corticosteroid, *RCC* relative change in coefficient, *SMD* standardized mean difference, *yr* during the baseline year

^a^*P* values shown using Kruskal–Wallis equality-of-populations rank test or Pearson’s *χ*^2^-test of independent categories for continuous and categorical variables, respectively

^b^An SMD ≤ 10% indicates sufficient balance between groups. The baseline variables with RCC ≥ 2%, which we defined as indicating bias potential, were selected for the direct matching attempts

^c^38 (0.6%) and 69 (0.6%) patients in LABD and ICS groups were receiving maintenance OCS (see Supplementary Table 6)

^d^Antibiotics were those prescribed on the same day as a lower respiratory consultation (identified by a Read code for a lower respiratory event). Moderate-to-severe exacerbations are defined in Methods section

The mean ages of the matched treatment groups were 68, 71, and 68 years in diabetes onset, diabetes progression, and osteoporosis onset cohorts, respectively. Patients in the diabetes progression cohort included a slightly higher percentage of men (59%, 66%, and 62%, respectively). Approximately half of patients in each study cohort had experienced one or more moderate-to-severe exacerbations in the baseline year, and in each ICS treatment group in the three study cohorts, 60% or 61% of patients in the 2011 GOLD A/B groups were prescribed ICS (see Tables 1–3).

### Length of baseline period and follow-up

The median baseline period before the first prescription of ICS or LABD ranged from 15.6 to 17.5 years in the two matched treatment groups of each study cohort, whereas median outcome periods after the index date ranged from 3.7 to 5.6 years, with the longer outcome periods in ICS treatment groups (Table 4).

**Table 4:** Available patient data: number of years before index date and during follow-up expressed as median (interquartile range)

	Diabetes onset cohort		Diabetes progression cohort		Osteoporosis onset cohort	
Years	LABD (*N* = 6540)	ICS (*N* = 11,430)	LABD (*N* = 324)	ICS (*N* = 480)	LABD (*N* = 7279)	ICS (*N* = 12,619)
Baseline	17.0 (8.7–31.9)	15.6 (8.0–29.6)	17.5 (9.3–31.8)	16.4 (9.5–33.9)	17.0 (8.6–31.8)	15.6 (8.0–29.6)
Outcome^a^	4.7 (2.7–5.2)	5.6 (3.6–8.1)	3.8 (2.8–5.3)	4.9 (3.1–6.8)	3.7 (2.7–5.2)	5.5 (3.6–8.1)

*ICS* inhaled corticosteroid, *LABD* long-acting bronchodilator

^a^Including the first 1.5 years when outcome events were not assessed

### Outcome event rates

Patients initiating ICS had a mean rate of diabetes onset of 1.25 vs. 1.05 diagnoses per 100 patient-years for the LABD initiators (online Supplementary Table 7). The mean rates of diabetes progression were 33.3 vs. 37.2 per 100 patient-years for ICS vs. LABD initiators. In the osteoporosis onset cohort, the rate of osteoporosis diagnosis was 0.70 vs. 0.66 diagnoses per 100 patient-years, respectively.

### ICS vs. LABD initiation: all patients and by GOLD group

**Fig. 1:**
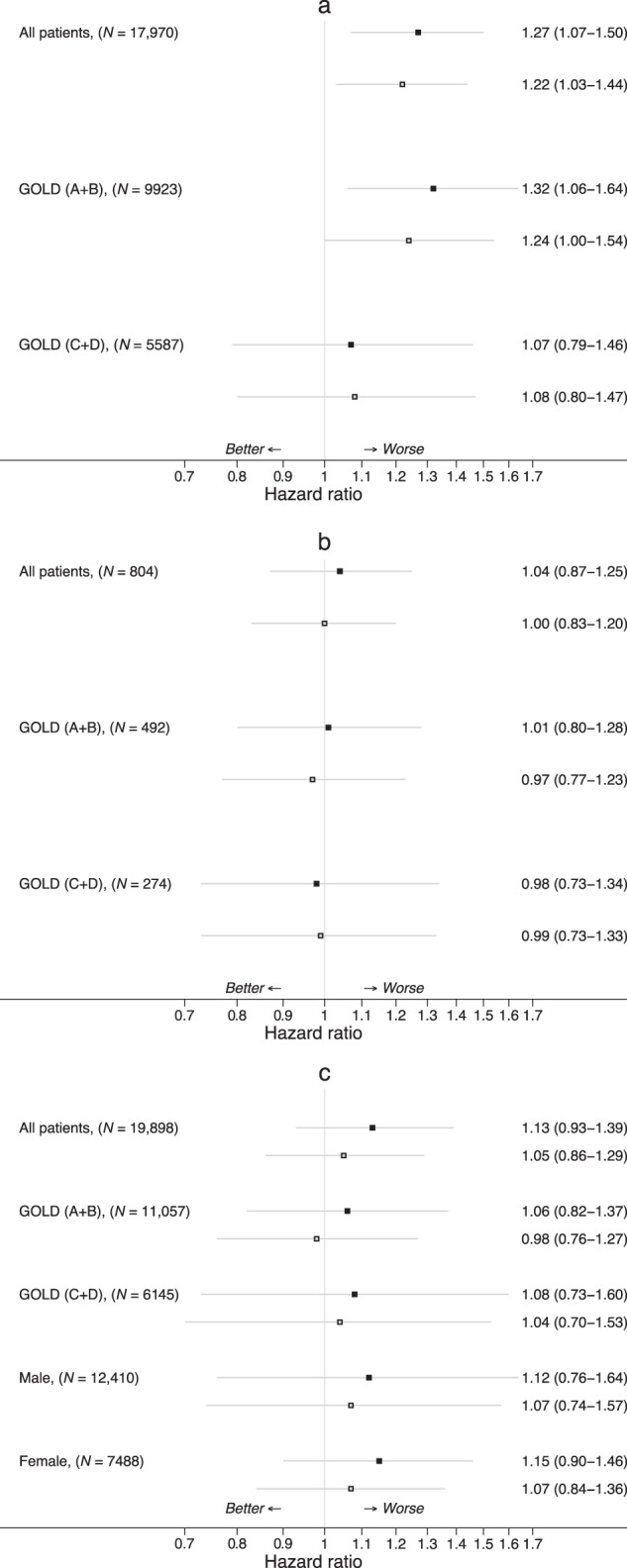
Hazard ratios (95% CIs) for matched inhaled corticosteroid (ICS) vs. long-acting bronchodilator (LABD) initiators, all patients and stratified by GOLD group, for **a** diabetes onset, **b** diabetes progression, and **c** osteoporosis onset (See Methods section for lists of variables used for adjustment in the outcome models.)

Results of the Cox proportional hazards models indicated that patients in the ICS group were more likely to develop type 2 diabetes compared with patients in the LABD group, with adjusted hazard ratio (HR) 1.27 (95% CI, 1.07–1.50; *P* = 0.006). The results were similar, i.e., increased risk of diabetes onset, in the subgroup of patients with mild to moderate disease severity (GOLD A/B), but not in the GOLD C/D subgroup (Fig. 1a).

The risk of diabetes progression was not significantly increased with ICS vs. LABD initiation, either among all matched patients, with HR 1.04 (0.87–1.25; *P* = 0.67), nor in GOLD A/B or GOLD C/D subgroups (Fig. 1b).

There was a non-significantly increased risk for osteoporosis onset among the ICS initiators compared with LABD initiators, with HR 1.13 (0.93–1.39, *P* = 0.22), with similar findings among GOLD A/B and C/D subgroups and among women and men analyzed separately (Fig. 1c).

### Outcomes by mean daily ICS exposure

**Fig. 2:**
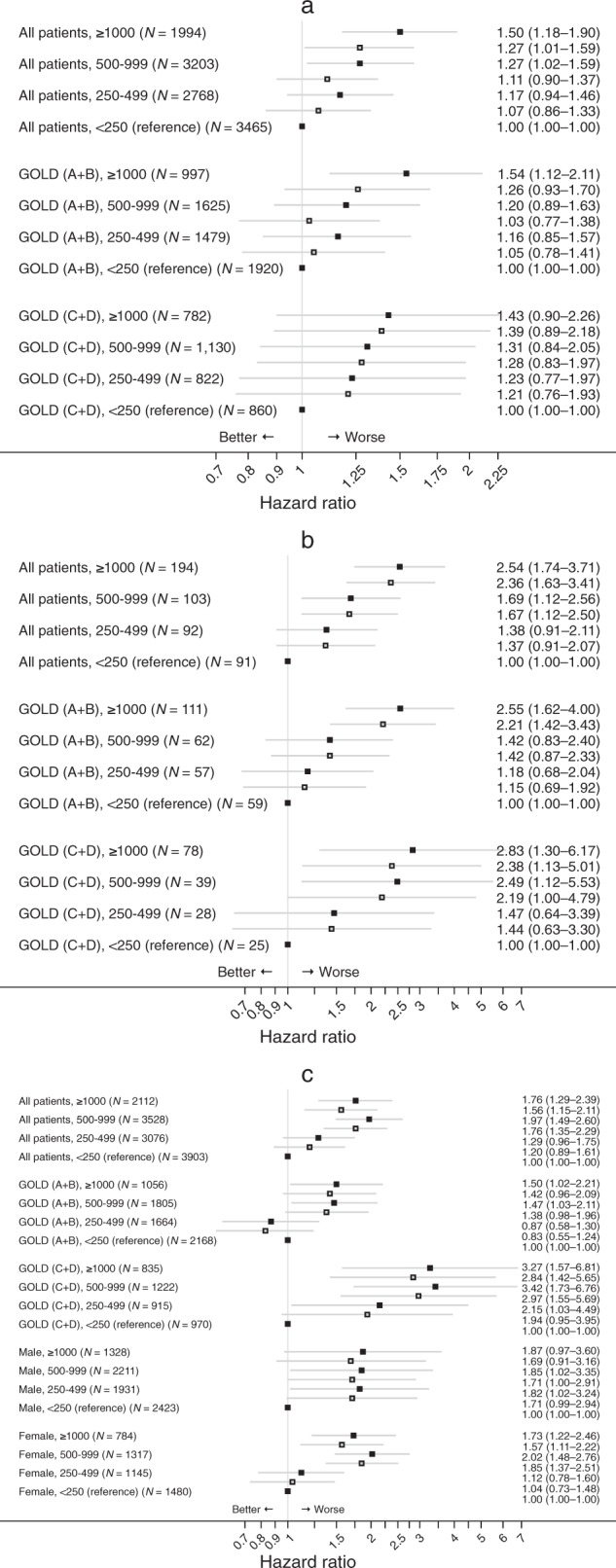
Hazard ratios (95% CIs) for mean daily inhaled corticosteroid (ICS) exposure (µg/day, vs. reference value of < 250 µg/day), all patients and stratified by GOLD group, for **a** diabetes onset, **b** diabetes progression, and also stratified by sex for **c** osteoporosis onset. (See Methods section for lists of variables used for adjustment in the outcome models)

The risk of diabetes onset showed a dose–response for mean daily ICS exposure, with significantly increased risk at mean daily exposures of ≥ 500 µg/day (compared with < 250 µg/day, fluticasone propionate–equivalent). A dose–response was evident in both GOLD A/B and GOLD C/D groups, with significantly increased risk of diabetes onset in GOLD A/B at exposures of ≥ 1000 µg/day (Fig. 2a).

The risk of diabetes progression also showed a clear dose–response relationship with mean daily ICS exposure for all patients and for the GOLD A/B and GOLD C/D subgroups, with significant increase in risk of diabetes progression for all patients and the GOLD C/D subgroup at exposures of ≥ 500 µg/day and for the GOLD A/B subgroup at exposures of ≥ 1000 µg/day (Fig. 2b).

We found that the risk of osteoporosis onset also increased with a clear dose–response for mean daily ICS exposure and significantly increased risk at exposures of ≥ 500 µg/day, with a similar pattern for the two GOLD subgroups and for women. Among men, all mean daily exposures were associated with increased risk that was statistically significant except for the greatest mean daily exposure (≥ 1000 µg/day; Fig. 2c).

### Outcomes by cumulative ICS exposure

There was no significant increase in adjusted results for risk and no consistent dose–response effect related to cumulative ICS exposure for diabetes onset, diabetes progression, or osteoporosis onset, either overall (online Supplementary Figs 2–4) or by GOLD group (data not shown). The results for diabetes progression showed lower precision, likely because of fewer patients in the higher cumulative exposure categories (online Supplementary Fig. 3).

## Discussion

The results of this large historical cohort study indicate that ICS initiation as the first maintenance treatment for patients with COPD is associated with greater risk of developing type 2 diabetes mellitus as compared with LABD treatment. There was no conclusive evidence to suggest the same association for diabetes progression or onset of osteoporosis overall (i.e., when all ICS doses were considered). However, for patients initiated on ICS, there were evident dose–response relationships for all three outcomes with regard to mean daily ICS exposure. We observed significantly greater risk of diabetes onset, diabetes progression, and osteoporosis onset at mean daily exposures of ≥ 500 µg/day in fluticasone propionate-equivalents (compared with reference value of < 250 µg/day). A dose–response relationship was not evident for cumulative ICS exposures. Importantly from a clinical perspective, there was considerable use of ICS outside of current GOLD recommendations, with over half of patients in 2011 GOLD groups A/B prescribed ICS.

We examined the relationship between ICS use and risk of side effects in a large COPD population, excluding those with asthma because ICS therapy for asthma is not contested: ICS are the cornerstone of asthma pharmacologic treatment and their wide-reaching benefits are well documented.^19^ Instead the benefits of ICS for patients with COPD, particularly those with milder disease (GOLD A/B), and particularly in comparison with LABDs, remain in question.^3,20,21^ Our findings place further doubt with regard to the risk-benefit ratio for ICS in COPD. Patients with COPD are already at increased risk of hip fracture, and they are older, hence more likely to have diabetes, osteopenia, or other comorbidities.^2,22^

Our findings with regard to diabetes onset in these large COPD cohorts extend earlier observational study findings in which patients with COPD were in the minority (only 17% and 22% of cases and controls, respectively, were considered to have probable COPD).^7^ In that study of patients treated for respiratory disease (asthma and COPD) in 1990 through 2007, a significant 34% increase in rate of diabetes onset was associated with current use of ICS. In addition, patients who were prescribed oral antidiabetic agents experienced a significant 34% increase in rate of diabetes progression to insulin therapy associated with ICS therapy.^7^ Instead, our findings did not reach statistical significance for the comparison between ICS and LABD for diabetes progression, for which post hoc power calculations indicated slightly lower analytical power than for the other comparisons. However, similar to the earlier findings, our study detected a dose–response effect of mean daily ICS exposure for diabetes progression as well as onset.

With regard to osteoporosis onset, prior studies of ICS therapy for COPD have used bone mineral density or fracture as outcomes.^11,12,23,24^ Instead, we elected to study onset to the first recorded diagnosis of osteoporosis, a diagnosis that is associated with increased risk of fracture. The lack of significantly increased risk when comparing ICS use with LABD use may be of limited relevance, as the ICS group was populated with patients with a mixture of exposure levels, which may have diluted the real effect of ICS exposure on the onset of osteoporosis. Indeed, we observed clear evidence of dose–response on the onset of osteoporosis for mean daily ICS exposure, beginning at ≥ 500 µg/day (vs. <250 µg/day) for all patients and for both women and men. However, the rationale for the diagnosis of osteoporosis (e.g., dual-energy x-ray absorptiometry vs. fragility fracture) was not documented, thus the clinical significance of the diagnosis may vary based on how the disease was revealed. Conversely, patients could have had undiagnosed osteoporosis, leading to an underestimation of ICS effects on osteoporosis onset.

Interestingly, we observed the adverse effects of ICS therapy in relation to mean daily exposure but not cumulative exposure. Corticosteroids increase the risk of diabetes in part by increasing insulin resistance, which allows blood glucose levels to rise;^25^ and corticosteroids affect bone turnover by enhancing bone resorption and decreasing bone formation.^26^ Significant decreases in levels of osteocalcin, a marker of bone formation, have been recorded in patients with COPD prescribed long-term therapy with inhaled beclomethasone dipropionate.^27^ These systemic effects would be more likely at the higher systemic exposures that occur at higher doses of ICS, as evidenced by studies demonstrating systemic effects and dose–response of systemic effects with ICS administration.^21,28–30^ We hypothesized that the mean daily exposure reflected the real intensity of treatment (i.e., resulting in effects on metabolism and bone formation), as opposed to high cumulative exposure, which could be the result of long-term but low daily exposures, hence resulting in fewer systemic effects. Indeed, the relation between cumulative exposure and onset of diabetes or osteoporosis was the reverse of what was expected: the greatest ICS cumulative exposure stratum showed the lowest rates of diabetes and osteoporosis onset. This is likely owing to inverse causation, as the cumulative exposure measure has a function of time incorporated. The patients with longer follow-up have lower likelihoods to develop the disease but they accumulate ICS.

We believe this study has several important strengths relative to prior work, including both prior observational studies and randomized controlled trials. We employed two large well-regarded databases, tapping into big numbers of patients and data over a long time period that enabled us to identify true initiators of therapy for COPD (i.e., with long baseline periods without COPD therapy) and to follow them for several years, much longer than possible in most randomized controlled trials. The coding of COPD is reliably recorded in the databases, especially since 2004 and the start of the UK Quality Outcomes Framework, which is when most of our study patients initiated COPD maintenance therapy.^31^ We employed multiple approaches for handling confounding by combining direct matching with a propensity score, plus adjustment for residual confounding after matching as well as adjustment for time-varying exposure to oral corticosteroids (OCS). Although we did not specifically exclude patients who were on maintenance OCS, we did indeed exclude any patient prescribed five or more OCS prescriptions during any study year, which effectively excludes maintenance OCS therapy as most UK prescriptions are for 1 month of treatment. Our rationale for minimizing the inclusion of patients on maintenance OCS in the study was because statistical adjustments, even with time-varying exposure measures, have their limitations. Therefore, we covered this possible confounding effect of OCS by both precise statistical adjustment, as well as by restriction to fewer than five OCS courses per year.

Using electronic medical record data instead of claims data provided the high granularity of information that enabled us to model three aspects of ICS exposure, including ICS vs. LABD, mean daily ICS exposure, and cumulative exposure. Moreover, the mean daily exposure variable served to support adherence with ICS therapy as it was calculated using prescribing data, considered reliable in the CPRD.^32^ Finally, we studied a patient population representative of patients seen in routine clinical practice rather than those enrolled in randomized controlled trials, which tend to include younger patients and more men than women.^33^ Approximately half of patients in our study had experienced a COPD exacerbation during the baseline year, similar to the 53% reported in a recent study of the natural history of COPD exacerbations.^34^

Median follow-up times in each study group were over 3.5 years and tended to be longer in the ICS group than the LABD group of each study cohort (4.9–5.6 vs. 3.7–4.7 years, respectively). Patients prescribed ICS could receive concomitant LABD and remain in the outcome analyses. However, follow-up ended for patients in the LABD group if they were prescribed ICS, and one could speculate that patients initiated on ICS may have exacerbated, also needing OCS, although we did not follow them beyond the time of ICS initiation. In a prior study,^16^ a big driver of therapy change was exacerbations, which could explain the relatively long follow-up in each treatment group of each study cohort because approximately two-thirds of patients with available GOLD group data in each group were in GOLD A or B.

A study limitation is that the databases include information collected for clinical use rather than specifically for research purposes, and some information is not reliably included, such as hospitalizations and emergency department visits.^35^ Moreover, the diabetes progression cohort was relatively small. One study found that rates of diabetes and musculoskeletal conditions were underestimated in the CPRD;^36^ however, we do not expect this under-registration to be different for the ICS and LABD treatment groups, and therefore comparative effects should not be biased. Another factor that could have led to an underestimation of ICS effects is the decision to adopt the conservative approach of analyzing data beginning at 1.5 years after ICS/LABD initiation.

It would have been of interest to examine diabetes onset during that 1.5-year period, and this remains a topic for further study. Moreover, analyses excluding patients who received any OCS prescription, as well as analyses examining OCS safety in COPD, are of great interest for future work.

As for all observational studies there remains the potential for unmeasured confounding. Our analyses were limited to the available data, which precluded the inclusion of some potential confounding factors, such as socioeconomic status, physician characteristics, and details of disease severity, including spirometry results. Although the level of patients’ physical activity, a driver of both type 2 diabetes and osteoporosis, was not available, we note that Medical Research Council dyspnea scale scores were available for most patients and were not significantly difference between treatment cohorts. Finally, another limitation could relate to the long timespan of the study, which is likely associated with time-related heterogeneity of patients’ clinical characteristics and treatments. However, the impact of this heterogeneity is limited by the matching process.

The proportions of patients in GOLD A/B groups prescribed ICS (60% or more) was similar to other studies in the United Kingdom.^16,17^ Discrepancies between COPD treatment recommendations and prescribing practices for ICS in COPD have been described also from other countries in Europe and North America.^15,37^ We would note, however, that we are using 2011 GOLD groups to categorize prescribing practices dated predominantly from 2004–2011. A recent study reports that by 2015 in the UK, ICS-containing first maintenance therapy for 2011 GOLD A/B COPD had declined to 47%;^17^ in addition, ICS monotherapy prescriptions have declined with time in the UK, and long-acting muscarinic antagonists (LAMA) prescriptions have increased with time.^16^ (The first LAMA was licensed in the UK in 2002).

We observed a clear dose–response relationship between mean daily exposure to ICS and increased risk for diabetes onset, diabetes progression, and osteoporosis onset for patients with COPD in this large matched cohort study. Long-term ICS therapy for COPD at mean ICS exposures of 500 µg/day or greater (vs. < 250 µg/day, fluticasone propionate-equivalent) is associated with an increased risk of diabetes, diabetes progression, and osteoporosis. In addition, we found considerable use of long-term medium- to high-dose ICS therapy outside of current GOLD strategy recommendations, prescribing practices that expose patients to increased risks for serious adverse effects. Our findings support the importance of careful selection of COPD therapies and prescribing ICS only when indicated and at the lowest possible doses. Moreover, these findings support current GOLD recommendations for prescribing ICS selectively to patients with frequent COPD exacerbations despite optimal bronchodilator therapy.

## Methods

### Data sources

This historical matched cohort study used data from the Clinical Practice Research Datalink (CPRD) and the Optimum Patient Care Research Database (OPCRD), two large, well-managed UK databases containing anonymized, longitudinal medical record data drawn from general practices, which serve as central locations for medical records in the UK.^38,39^ The CPRD contains medical record data for about five million patients from over 600 subscribing practices and has long been used for pharmacoepidemiological research.^32,39,40^ The OPCRD is a database developed to improve patient outcomes through medical research and services, with focus on patient-reported outcomes, that, at the time of this study, contained anonymous data for over 2.4 million patients from over 576 primary care practices across the United Kingdom.^38^ The OPCRD has been reviewed and ethically approved by the NHS Health Research Authority to hold and process anonymized data as part of service delivery (Research Ethics Committee reference: 15/EM/0150). We used Quality Outcome Framework (QOF) diagnostic Read codes, which are part of the UK national quality improvement initiative and pay-for-performance scheme. The diagnostic and prescribing information in the CPRD has been evaluated and is considered to be reliable, particularly since the introduction in 2004 of QOF, which provides incentives for practitioners to conduct diagnostic screening and recording for COPD and other diseases.^31^

The study was conducted according to recommendations for observational research.^41^ The protocol was approved by the CPRD Independent Scientific Advisory Committee (ISAC reference number 16_040) and the Anonymised Data Ethics Protocols and Transparency committee (ADEPT Approval Reference ADEPT1316), the independent scientific advisory committee for the OPCRD.^38^ The study was registered with the European Network Centres for Pharmacoepidemiology and Pharmacovigilance (ENCePP), European Union electronic Register of Post-Authorisation Studies (EU PAS Register number 13218).^42,43^ No patient identifying information was accessible during the study.

The CPRD and OPCRD data sets were constructed separately and checked for overlap, before pooling for analyses, in order to exclude duplicate patients. Identification of patients who were present in both data sets was conducted by matching on a number of variables, such as the year of birth, sex, and index date. During this process, patients were never identifiable and the data analysts who constructed the data sets had no access to any data in a patient identifiable form. The rationale for combining CPRD and OPCRD data sets was to increase the number of patients included in the study groups and therefore increase the power to detect clinically relevant differences between treatment cohorts. Together, the data sets comprised medical record information spanning from 1983 to May 2016.

### Study design and patients

**Fig. 3:**
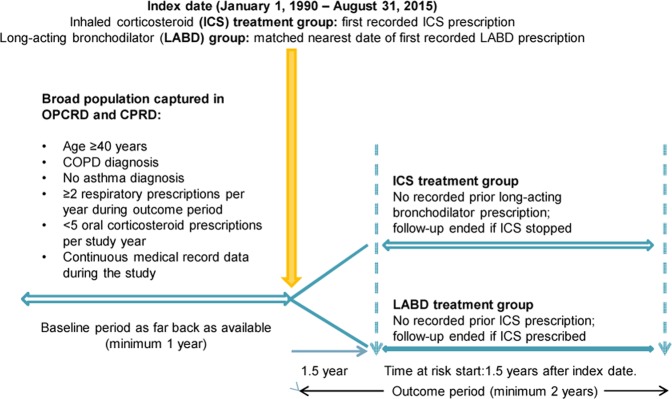
Study design. CPRD Clinical Practice Research Datalink, OPCRD Optimum Patient Care Research Database

Eligible patients had a record of physician-diagnosed COPD, were aged 40 years or older when prescribed their first ICS or LABD maintenance therapy for COPD, and had continuous medical records for a ≥ 1-year baseline period before the index date (first COPD maintenance therapy), followed by an outcome period of ≥ 2 years (Fig. 3). Patients were required to have no recorded diagnosis of asthma or to have an asthma-resolved code recorded after the asthma diagnostic code and before the index date. In addition, eligible patients had to receive two or more respiratory prescriptions (ICS or LABD, depending on treatment group) during each outcome year and be prescribed less than five OCS prescriptions during each study year. In the United Kingdom, the prescribed dose for an acute course of OCS is variable but prednisone 30–40 mg per day for 5 days is usual. The index date range was January 1990 to August 2015.

Patients were assigned to the ICS or LABD treatment group according to their first COPD prescription and were followed from the index date to the first outcome event, the end of available data, the end of ICS use (ICS group) or the initiation of ICS (LABD group), whichever occurred first. Patients initiating ICS (the ICS group) had no previous prescriptions for LABA, LAMA, or combination LABA/LAMA and had received two or more ICS prescriptions during at least 2 outcome years. Prescribing of LABD was permitted in the ICS group, and the prescribed ICS drug and/or inhaler could change during the outcome period; the study observation period ended if ICS were stopped. Patients initiating LABD therapy as LABA, LAMA, or combination LABA/LAMA (LABD group) had no previous ICS prescriptions; the LABD drug(s) could change during outcome, and the study observation period ended if ICS were prescribed. Patients in the ICS and LABD groups were unique, i.e., patients did not contribute data to both cohorts.

We compared outcomes for the two treatment groups within three unique study cohorts with specific eligibility criteria designed to capture: (1) the onset of diabetes (diabetes onset cohort), (2) the progression of diabetes (diabetes progression cohort), and (3) the onset of osteoporosis (osteoporosis onset cohort). Eligibility criteria specific to each cohort are summarized in Table 5. The diabetes onset cohort included patients with no prior recorded type 2 diabetes mellitus diagnosis and/or antidiabetic treatment, and not more than one HbA1c reading of > 6.5%, ever before the index date or within 1.5 years after the index date. A prior diagnosis of type 1 diabetes mellitus ever before the index date was also cause for exclusion. Patients prescribed metformin for physician-diagnosed polycystic ovary syndrome were not excluded. The diabetes progression cohort included patients with recorded diagnosis and/or treatment for type 2 diabetes mellitus and/or two or more HbA1c readings > 6.5% ever before the index date. In addition, included patients had one or more HbA1c recorded readings in both the baseline year and the outcome period starting at 1.5 years after the index date. Exclusion criteria for the diabetes progression cohort were a recorded diabetes-resolved code after the diabetes diagnostic code, physician-diagnosed type 1 diabetes mellitus ever before the index date, and physician-diagnosed polycystic ovary syndrome with one or more metformin prescriptions before the index date. The osteoporosis onset cohort included patients with no prior recorded osteoporosis diagnosis ever before the index date or within 1.5 years after the index date.

**Table 5:** Key eligibility criteria and outcome definitions specific to each of the three study cohorts

1. Diabetes onset cohort
Exclusion criteria:
Prior recorded type 2 diabetes mellitus diagnosis^a^ and/or antidiabetic treatment and/or two or more HbA1c readings of > 6.5%, ever before or within 1.5 years after the index date
Diagnosis of type 1 diabetes mellitus ever before the index date
Outcome definition of diabetes onset:
Diagnosis of type 2 diabetes mellitus and/or
Antidiabetic drug prescription(s), and/or
At the time of the second of two or more HbA1c readings of > 6.5%
2. Diabetes progression cohort
Inclusion criteria:
Diagnosis and/or treatment for type 2 diabetes mellitus and/or ≥2 HbA1c readings >6.5% ever before the index date
One or more HbA1c readings in both the baseline year and the outcome period starting at 1.5 year after the index date
Exclusion criteria:
Recorded diabetes-resolved code after the diagnostic code, diagnosis of type 1 diabetes mellitus ever before the index date, and diagnosis of polycystic ovary syndrome with one or more metformin prescriptions ever before the index date
Outcome definition of diabetes progression (includes worsening disease control)
Increase in HbA1c readings of 0.5% and greater from baseline to outcome period, and/or
Prescription for an increase in daily dose of glucose-regulating drug, excluding increases at the index date or within 3 months of the index date, and/or
Addition of a new class of glucose-regulating drug without another class being discontinued, and/or
Progression of treatment to insulin from index date to outcome period, in patients without insulin prescriptions in baseline
3. Osteoporosis onset cohort
Exclusion criterion:
Prior recorded osteoporosis diagnosis ever before the index date or within 1.5 year after the index date
Outcome definition of osteoporosis onset
Diagnosis of osteoporosis

^a^Diagnoses were defined as recorded diagnostic Read codes. All code lists are available on request

We categorized patients into 2011 GOLD groups A, B, C, or D^18^ using number of moderate-to-severe exacerbations (at least 15 days apart) during the baseline year together with values recorded closest to and within 5 years before the index date for the Medical Research Council (MRC) dyspnea scale (scored from 1 to 5, with 5 representing the worst dyspnea) and forced expiratory volume in 1 s (FEV_1_). Exacerbations during the baseline year were defined as occurrence of COPD-related unscheduled hospital admission or accident & emergency attendance; or prescription of an acute course of OCS; or prescription of antibiotics on the same day as a lower respiratory consultation (identified by a Read code for a lower respiratory event). Hence the GOLD groups were defined as follows:

- GOLD A: MRC score 1–2 and FEV_1_ ≥ 50% and ≤ 1 COPD exacerbation with no hospitalization required for COPD.
- GOLD B: MRC score ≥ 3 and FEV_1_ ≥ 50% and ≤ 1 COPD exacerbation with no hospitalization required for COPD.
- GOLD C: MRC score 1–2 and FEV_1_ < 50% and/or ≥ 2 COPD exacerbations or ≥ 1 exacerbation leading to hospital admission.
- GOLD D: MRC score ≥ 3 and FEV_1_ < 50% and/or ≥ 2 COPD exacerbations or ≥ 1 exacerbation leading to hospital admission.

### Study endpoints

The exposures of interest were (1) ICS as compared with LABDs, (2) mean daily ICS exposure (µg/day, all prescribed ICS divided by number of follow-up days), and (3) cumulative ICS exposure (mg, total dose over the follow-up period).

Time at risk of an outcome event was assessed beginning at 1.5 years after the index date.^8^ Patients with diabetes onset or osteoporosis onset occurring within 1.5 years after the index date were excluded from the respective study cohorts, and diabetes progression measures were not counted in that time frame for the diabetes progression cohort based on the clinical decision that cases within 1.5 years of index date could not be attributed to ICS initiation and would therefore bias the conclusions.

Onset of diabetes was defined as type 2 diabetes mellitus diagnosis and/or antidiabetic drug prescriptions, and/or two or more HbA1c readings > 6.5%. Patients with physician-diagnosed polycystic ovary syndrome and one or more metformin prescriptions in the outcome period were excluded from the analysis.

Diabetes progression was defined as worsening disease control or treatment changes indicating disease progression. Worsening disease control was measured by change in blood glucose level, defined as increased HbA1c readings of 0.5% and higher, considered to be a clinically significant change.^44,45^ Change in HbA1c from the baseline year to the first HbA1c reading after 1.5 years after the index date was compared between the ICS and LABD groups. Disease progression was defined as any of three types of treatment change:

1. Dose increase of glucose-regulating drug prescription: defined as an increase in the prescribed daily dose of any glucose-regulating drug prescribed to the patient. Dose increases at the index date or within 3 months of the index date were not considered to be disease progression because physicians may increase the dose of glucose-regulating drugs owing to the known impact of initiating corticosteroids on glucose levels.
2. Addition of a glucose-regulating drug class: defined as a new drug class started without another class being discontinued. Oral glucose-regulating drugs were categorized into six classes: (i) sulfonylureas, (ii) metformin, (iii) acarbose, (iv) thiazolidinediones, (v) dipeptidyl peptidase 4 (DPP-4) inhibitors, and (vi) other oral drugs.
3. Progression of treatment to insulin: time between index date and first prescription of insulin in outcome period, in patients without insulin prescriptions in baseline.

Osteoporosis onset was defined as a diagnostic Read code for osteoporosis. Osteoporosis drug prescriptions without an accompanying diagnostic code were not considered indicative of osteoporosis because commonly prescribed osteoporosis drugs are prescribed for osteopenia and for other conditions (e.g., bisphosphonates for patients with Paget’s disease, bone metastasis, or multiple myeloma and strontium for patients with certain forms of cancer).

We also conducted per-protocol comparisons among different ICS formulations and inhalers; however, the comparisons were underpowered and uninterpretable, hence are not reported here.

### Cohort matching

We matched patients in the ICS and LABD treatment groups using a mixed matching process at ratios of 1:1 to 3:1. Patients were matched on a set of baseline characteristics identified and predefined by expert judgment as well as a propensity score created with baseline variables showing relevant bias, evaluated using the standardized mean difference (SMD)^46^ in combination with the bias potential.^47^

First, a characterization of all baseline demographics, comorbidities, indicators of disease severity, and other patient-related variables was carried out for each treatment group within each study cohort. The difference between treatment groups was quantified using the SMD.^46^ This measure is not affected by the number of observations and is thus a better way to judge imbalance than a *P* value of a hypothesis test of difference. It also provides insight into the magnitude of the difference. The SMD was calculated for both continuous and categorical variables. An SMD of ≤ 10% was judged to indicate sufficient balance between the treatment (ICS) and reference (LABD) groups.

Bias potential was measured using the relative change in coefficient (RCC), also known as the change-in-estimate, of the exposure when the covariate was added into the model predicting outcome. Bias potential assesses the degree to which the observed association between the exposure of interest and the outcome is affected by conditioning on the variable.^47,48^ The baseline variables with RCC ≥ 2%, which we defined as indicating bias potential, were selected for the direct matching attempts.

Missing data were treated as missing completely at random and were not imputed. Variables with > 10% of missing data were not considered for matching and/or the generation of the propensity score. Variables with missingness of ≤ 10% were encoded into categorical variables, adding a category for the observations with missing values, enabling these variables to be used for matching and/or propensity score generation.

Exact matching for categorical variables and matching within a maximum caliper (maximum distance allowed between a case and a control) for continuous variables was used to match patients using nearest neighbor variable mixed matching with a match maximum of 3:1 without replacement. Mixed matching is a process that helps to utilize more of the data by matching varying numbers of patients in the control group to each patient in the treatment group. In other words, there was a group of unique patients matched 1:1, another group of unique patients matched 1:2, and a third group of unique patients matched 1:3. The analysis was conducted using all of the matched patients although some patients had one match whereas other patients had three matches.

The following variables (±their caliper) were used for matching:

1. Year of index date ± 2 years
2. Age ± 5 years
3. Sex
4. Smoking status
5. BMI category (except diabetes progression cohort)
6. Number of exacerbations (recorded as Read code) in the baseline year, categorized

Attempted direct matching in the diabetes progression cohort found that only 50% of patients were matchable with the direct matching criteria; therefore, BMI was not used for direct matching in that cohort but was instead included as part of the propensity score.

The propensity score was generated using a logistic regression model with the variables listed in Table 6. The caliper used for the propensity score during matching was 0.25 times its standard deviation.^49^

**Table 6:** Variables used to generate the propensity score

Age (years)
Sex
Smoking status
Body mass index (kg/m^2^)
Cardiovascular disease diagnosis
Ischemic heart disease diagnosis
Hypertension diagnosis
Components of the Charlson comorbidity index, diagnoses of:
Myocardial infarction
Stroke
Heart failure
Connective tissue disorders
Dementia
Diabetes
Mild liver disease
Peptic ulcer
Peripheral vascular disease
Pulmonary disease
Cancer
Paraplegia
Other chronic respiratory diseases
Number of prescriptions (categorized):
Nasal corticosteroids
Antibiotics prescribed on the same day as a lower respiratory consultation (identified by a Read code for a lower respiratory event)
Acute oral corticosteroids (OCS)
All OCS
Short-acting muscarinic antagonists (SAMA)
Medication ≥ 1 prescriptions (yes/no):
Maintenance OCS
Long-acting β-agonists (LABA)
Long-acting muscarinic antagonists (LAMA)
Methylxanthines
Leukotriene receptor antagonists
Short-acting β-agonists (SABA), mean daily dose (salbutamol equivalents), categorized
Number of (categorized):
Exacerbations (moderate/severe)
Emergency department respiratory attendances
Inpatient respiratory admissions
Outpatient respiratory visits

For each outcome cohort, 20 matching runs with different random patient orders were made to select the best combination of number of matched patients and multivariable balance statistics. The statistics used were as follows:

- Number of cases (ICS initiators) matched.
- C-statistics of the prediction model of exposure using all variables with < 10% missing values, in the matched. This is a measure of discriminative ability of the set of 35 baseline characteristics for exposure.
- Percentage of variables of the 35 baseline characteristics used to generate the propensity score that achieved residual bias potential < 1 and < 0.5%.

### Statistical analyses

For tests of differences at baseline, we used the Kruskal–Wallis equality-of-populations rank test and Pearson’s *χ*^2^-test of independent categories for continuous and categorical variables, respectively.

We used conditional proportional hazards regression, adjusting for residual confounding, to compare ICS vs. LABD and to model ICS exposures for time-to-event outcomes. Mean effects and their 95% confidence intervals (CIs) and two-sided *p* values were calculated for all patients and stratified by 2011 GOLD groups A/B and C/D.^18^

We adjusted for variables with residual confounding (causing at least 2% RCC). In addition, adjusted models also included age, sex, and two time-varying covariates representing exposure to OCS over time. For each 365 days of the follow-up period, the number of OCS prescriptions was calculated, as well as the cumulative number of OCS prescriptions until that follow-up year.

Assumptions for regression models were assessed as appropriate.

Additional variables used for adjustment in the outcome models comparing matched ICS vs. LABD treatment groups were as follows:

- For the diabetes onset cohort: number of OCS prescriptions;
- For the diabetes progression cohort: number of moderate-severe exacerbations and years since type 2 diabetes diagnosis;
- For the osteoporosis onset cohort: none.

Within the ICS group only, we analyzed mean daily ICS exposure compared with the reference value of < 250 µg/day in fluticasone propionate-equivalents and cumulative ICS exposure compared with reference value of < 50 mg in fluticasone propionate-equivalents. The within-ICS treatment group comparisons (split by ICS mean daily exposure and by ICS cumulative exposure) were restricted to the patients who were matched to LABD group patients in order to facilitate comparisons of results with those from the ICS vs. LABD comparisons. Mean daily exposure was calculated in µg as the cumulative amount of ICS prescribed, divided by number of days since ICS initiation; cumulative ICS exposure was calculated in mg as the total dose prescribed over the follow-up period. Mean daily and cumulative ICS exposures were expressed in fluticasone propionate–dose equivalents, as follows: doses of fluticasone propionate, ciclesonide, and extrafine-particle beclomethasone were reported as actual doses and budesonide and fine-particle beclomethasone dipropionate doses were halved.^50^ Both mean daily ICS exposure and cumulative ICS exposure were modeled as time-varying variables, meaning that at the time of each new ICS prescription, the mean daily ICS dose and the cumulative ICS dose up to that moment in time were updated.

We used the variables with at least 2% RCC (bias potential) for the ICS group-only exposures for adjustment in the outcome models:

ICS mean daily exposure:

- For the diabetes onset cohort: none;
- For the diabetes progression cohort: number of acute OCS prescriptions, glucose-regulating medication use;
- For the osteoporosis onset cohort: number of SAMA prescriptions.

ICS cumulative exposure:

- For the diabetes onset cohort: none;
- For the diabetes progression cohort: number of acute OCS prescriptions, number of nasal corticosteroid prescriptions, number of SAMA prescriptions;
- For the osteoporosis onset cohort: number of SAMA prescriptions.

In the final matched data sets all variables with ≤ 10% missing values in each treatment group and with RCC of at least 2% were identified for adjustment in the outcome models. We expected that these variables could have similar associations with exposure and/or outcome; therefore, we assessed their conditional bias relative to the variables already in the model. Starting with a model with exposure as the only explanatory variable, we added additional variables one by one in order of their individual bias potential, highest first. After a variable was added to the model it was retained if it caused a change-in-estimate of at least 2% relative to the prior model.

Post hoc power calculations for the matched cohorts determined that we had 80% power to show the following minimal HR for the ICS vs. LABD comparisons:

- HR = 1.10 for diabetes onset,
- HR = 1.16 for diabetes progression, and
- HR = 1.11 for osteoporosis onset.

Analyses were conducted using Stata MP version 12 and Stata SE version 14 (StataCorp, College Station, TX). Statistically significant results were predefined as *P* < 0.05.

## Supplementary information

Supplementary file

## Data Availability

The data sets supporting the conclusions of this article were derived from the Clinical Practice Datalink (http://www.cprd.com) and the Optimum Patient Care Research Database (http://opcrd.co.uk/). We do not have permission to give public access to these data sets; however, researchers may request access for their own purposes.
