# Supplementary material for: Inhaled corticosteroids in COPD and onset of type 2 diabetes and osteoporosis: matched cohort study
Source: NPJ Prim Care Respir Med. 2019 Oct 28;29:38. doi: 10.1038/s41533-019-0150-x (PMC6817865; doi:10.1038/s41533-019-0150-x)

Supplementary Information

Inhaled corticosteroids in COPD and the onset of type 2 diabetes and osteoporosis: historical matched cohort study

Supplementary Fig. 1. Patient selection from the databases

OCS, oral corticosteroids

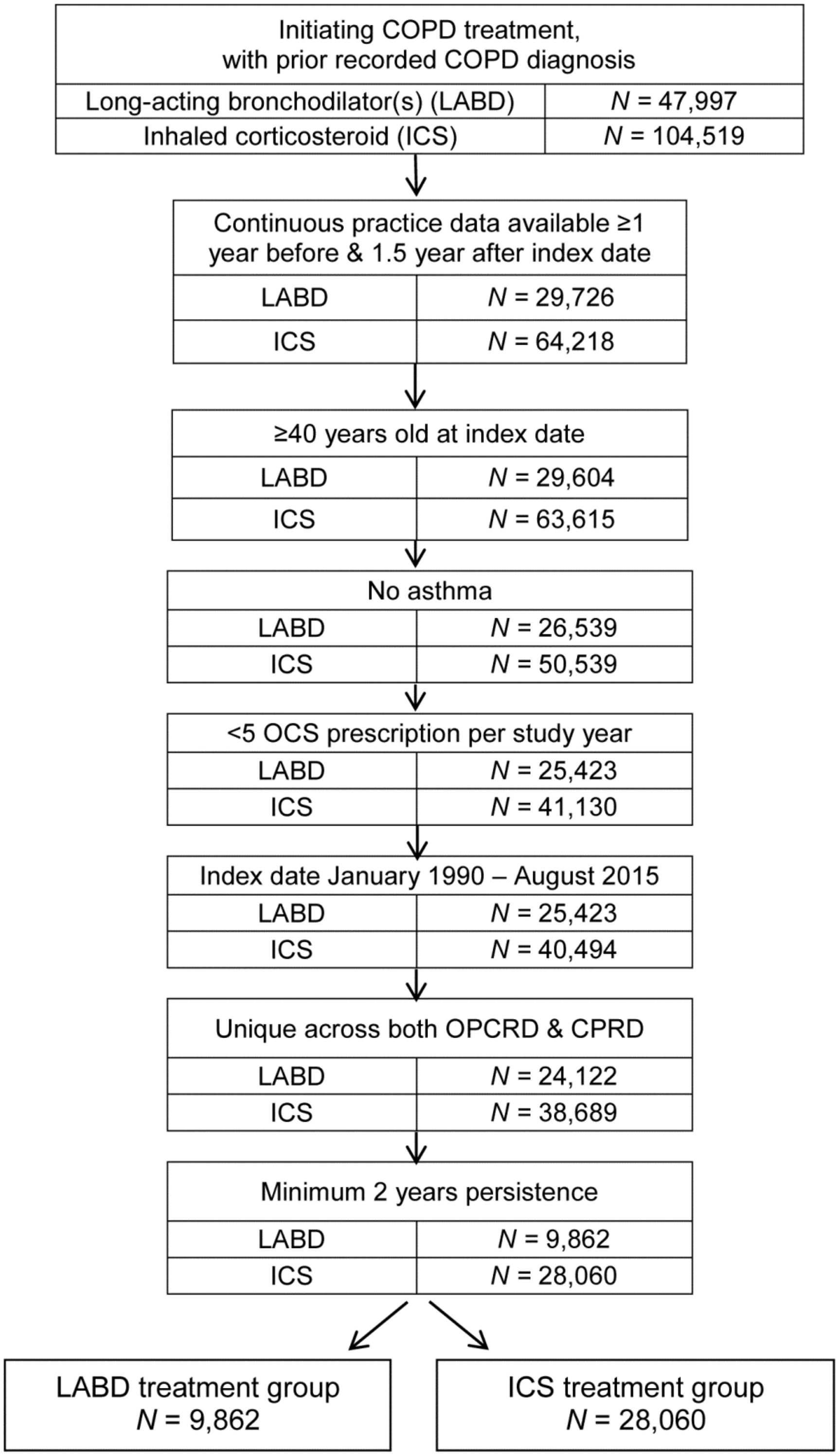

Supplementary Table 1. Baseline characteristics of diabetes onset cohort: unmatched

Variable		LABD (n = 8556)	ICS (n = 25,378)	P value*	SMD (%)†	RCC (%)†
Index year	N (% non-missing)	8556 (100.0)	25,378 (100.0)	<0.0001	110.5	5.6
	Mean (SD)	2008.2 (3.8)	2002.7 (5.9)			
	Median (IQR)	2009.0 (5.0)	2003.0 (9.0)			
Age (years)	N (% non-missing)	8556 (100.0)	25,378 (100.0)	<0.0001	6.4	1.1
	≥40 – <60 n (%)	1612 (18.8)	5432 (21.4)			
	≥60 – <80 n (%)	5747 (67.2)	16,672 (65.7)			
	≥80 n (%)	1197 (14.0)	3274 (12.9)			
Age (years)	Mean (SD)	68.4 (10.0)	67.9 (10.3)	0.0009	5.0	1.1
Sex	N (% non-missing)	8556 (100.0)	25,378 (100.0)	0.3041	1.3	0.2
	Male n (%)	4914 (57.4)	14,414 (56.8)			
Smoking status	N (% non-missing)	8403 (98.2)	23,446 (92.4)	<0.0001	6.7	4.3
	Never n (%)	521 (6.2)	2081 (8.9)			
	Current n (%)	3849 (45.8)	10,460 (44.6)			
	Ex-smoker n (%)	4033 (48.0)	10,905 (46.5)			
BMI (kg/m^2)	N (% non-missing)	8123 (94.9)	22,581 (89.0)	<0.0001	7.7	10.8
	<18.5 n (%)	390 (4.8)	1294 (5.7)			
	18.5 – <25 n (%)	3083 (38.0)	8997 (39.8)			
	25 – <30 n (%)	2736 (33.7)	7628 (33.8)			
	≥30 n (%)	1914 (23.6)	4662 (20.6)			
Years since COPD diagnosis	N (% non-missing)	8556 (100.0)	25,378 (100.0)	<0.0001	63.7	0.4
	>10 years n (%)	939 (11.0)	9357 (36.9)			
Cardiovascular disease	N (% non-missing)	8556 (100.0)	25,378 (100.0)	<0.0001	8.0	1.6
	Yes n (%)	3030 (35.4)	8028 (31.6)			
Ischemic heart disease	N (% non-missing)	8556 (100.0)	25,378 (100.0)	0.0004	4.3	1.1
	Yes n (%)	1680 (19.6)	4552 (17.9)			
Hypertension	N (% non-missing)	8556 (100.0)	25,378 (100.0)	<0.0001	15.8	3.9
	Yes n (%)	3148 (36.8)	7452 (29.4)			
Cancer	N (% non-missing)	8556 (100.0)	25,378 (100.0)	<0.0001	19.4	0.1
	Yes n (%)	1774 (20.7)	3418 (13.5)			
Charlson Comorbidity Index	N (% non-missing)	8556 (100.0)	25,378 (100.0)	<0.0001	4.6	0.2
	0–1 n (%)	7249 (84.7)	20,627 (81.3)			
	2–5 n (%)	590 (6.9)	2828 (11.1)			
	6–10 n (%)	434 (5.1)	865 (3.4)			
	11–20 n (%)	247 (2.9)	971 (3.8)			
	21–30 n (%)	34 (0.4)	84 (0.3)			
	31–50 n (%)	2 (0.0)	3 (0.0)			
Nasal corticosteroids	≥1 prescription	536 (6.3)	2395 (9.4)	<0.0001	12.0	0.6
Antibiotic prescriptions	N (% non-missing)	8556 (100.0)	25,378 (100.0)	0.0019	2.1	0.0
	0 n (%)	5126 (59.9)	15,251 (60.1)			
	1 n (%)	1975 (23.1)	5553 (21.9)			
	2 n (%)	888 (10.4)	2609 (10.3)			
	≥3 n (%)	567 (6.6)	1965 (7.7)			
Acute OCS prescriptions	N (% non-missing)	8556 (100.0)	25,378 (100.0)	<0.0001	13.2	0.4
	0 n (%)	6958 (81.3)	19,130 (75.4)			
	1 n (%)	1119 (13.1)	4414 (17.4)			

Supplementary Table 1. Baseline characteristics of diabetes onset cohort: unmatched

**Variable**		**LABD (n = 8556)**	**ICS (n = 25,378)**	**P value***	**SMD (%)†**	**RCC (%)†**
	≥2 n (%)	479 (5.6)	1834 (7.2)			
OCS maintenance prescriptions‡	N (% non-missing)	8556 (100.0)	25,378 (100.0)	0.0005	4.6	0.1
	Yes n (%)	47 (0.5)	240 (0.9)			
All OCS prescriptions	N (% non-missing)	8556 (100.0)	25,378 (100.0)	<0.0001	12.9	0.4
	0 n (%)	6925 (80.9)	19,002 (74.9)			
	1 n (%)	1121 (13.1)	4421 (17.4)			
	2 n (%)	336 (3.9)	1237 (4.9)			
	≥3 n (%)	174 (2.0)	718 (2.8)			
SABA, mean daily dose (salbutamol equivalents), μg/day	N (% non-missing)	8556 (100.0)	25,378 (100.0)	0.0182	2.3	0.1
	0 n (%)	3681 (43.0)	10,970 (43.2)			
	>0 – ≤200 n (%)	3059 (35.8)	8832 (34.8)			
	>200 – ≤400 n (%)	931 (10.9)	2675 (10.5)			
	>400 – ≤600 n (%)	356 (4.2)	1076 (4.2)			
	>600 n (%)	529 (6.2)	1825 (7.2)			
SABA inhaler devices	N (% non-missing)	8556 (100.0)	25,378 (100.0)	<0.0001	12.2	0.1
	0 n (%)	5526 (64.6)	14,478 (57.0)			
	1 n (%)	1052 (12.3)	3726 (14.7)			
	2 n (%)	507 (5.9)	2063 (8.1)			
	3 n (%)	261 (3.1)	901 (3.6)			
	≥4 n (%)	1210 (14.1)	4210 (16.6)			
SAMA prescriptions	N (% non-missing)	8556 (100.0)	25,378 (100.0)	<0.0001	8.3	0.2
	0 n (%)	6600 (77.1)	20,155 (79.4)			
	1 n (%)	493 (5.8)	1650 (6.5)			
	2 n (%)	607 (7.1)	1686 (6.6)			
	3 n (%)	595 (7.0)	1304 (5.1)			
	≥4 n (%)	261 (3.1)	583 (2.3)			
FEV_1 %predicted	N (% non-missing)	5202 (60.8)	7787 (30.7)	<0.0001	12.3	7.4
	<30 n (%)	200 (3.8)	413 (5.3)			
	30–49% n (%)	1121 (21.5)	2113 (27.1)			
	50–79% n (%)	3092 (59.4)	4119 (52.9)			
	≥80% n (%)	789 (15.2)	1142 (14.7)			
Moderate-severe exacerbations	N (% non-missing)	8556 (100.0)	25,378 (100.0)	<0.0001	8.0	0.0
	0 n (%)	4509 (52.7)	12,537 (49.4)			
	1 n (%)	2382 (27.8)	7138 (28.1)			
	≥2 n (%)	1665 (19.5)	5703 (22.5)			
ED respiratory attendances	N (% non-missing)	8556 (100.0)	25,378 (100.0)	0.3070	1.3	0.0
	≥1 n (%)	93 (1.1)	311 (1.2)			
Inpatient respiratory admissions	N (% non-missing)	8556 (100.0)	25,378 (100.0)	0.2506	1.5	0.0
	≥1 n (%)	179 (2.1)	585 (2.3)			
Outpatient respiratory visits	N (% non-missing)	8556 (100.0)	25,378 (100.0)	<0.0001	10.8	0.2
	≥1 n (%)	393 (4.6)	658 (2.6)			
MRC score	N (% non-missing)	7674 (89.7)	15,605 (61.5)	<0.0001	2.5	4.2
	1 n (%)	1069 (13.9)	1995 (12.8)			
	2 n (%)	3732 (48.6)	7911 (50.7)			
	3 n (%)	1949 (25.4)	3625 (23.2)			

Supplementary Table 1. Baseline characteristics of diabetes onset cohort: unmatched

Variable		LABD (n = 8556)	ICS (n = 25,378)	P value*	SMD (%)†	RCC (%)†
	4 n (%)	801 (10.4)	1694 (10.9)			
	5 n (%)	123 (1.6)	380 (2.4)			
GOLD risk group	N (% non-missing)	7674 (89.7)	15,605 (61.5)	<0.0001	6.9	4.1
	GOLD A	3242 (42.2)	6238 (40.0)			
	GOLD B	1856 (24.2)	3472 (22.2)			
	GOLD C	1559 (20.3)	3668 (23.5)			
	GOLD D	1017 (13.3)	2227 (14.3)			

*P values shown using Kruskal-Wallis equality-of-populations rank test or Pearson's χ^2 test of independent categories for continuous and categorical variables, respectively.

†An SMD ≤10% indicates sufficient balance between groups. The baseline variables with RCC ≥2%, which we defined as indicating bias potential, were selected for the direct matching attempts.

‡Patients prescribed maintenance OCS received 4 or fewer prescriptions in any given year, as per study eligibility criteria.

ED, emergency department; FEV_1, forced expiratory volume in 1 second; GOLD, Global Initiative for Chronic Obstructive Lung Disease; ICS, inhaled corticosteroid; IQR, interquartile range; LABD, long-acting bronchodilator; MRC, Medical Research Council dyspnea scale; OCS, oral corticosteroid; RCC, relative change in coefficient; SABA, short-acting β-agonist; SAMA, short-acting muscarinic antagonist; SMD, standardized mean difference; yr, during the baseline year.

Supplementary Table 2. Baseline characteristics of diabetes progression cohort: unmatched

Variable		LABD (n = 485)	ICS (n = 861)	P value*	SMD (%)†	RCC (%)†
Index year	N (% non-missing)	485 (100.0)	861 (100.0)	<0.0001	73.1	5.6
	Mean (SD)	2009.1 (3.0)	2006.4 (4.1)			
	Median (IQR)	2009.0 (5.0)	2007.0 (6.0)			
Age (years)	N (% non-missing)	485 (100.0)	861 (100.0)	0.7810	0.9	0.4
	≥40 – <60, n (%)	52 (10.7)	98 (11.4)			
	≥60 – <80, n (%)	354 (73.0)	613 (71.2)			
	≥80, n (%)	79 (16.3)	150 (17.4)			
Age (years)	Mean (SD)	70.8 (8.7)	71.1 (8.9)	0.45	3.6	0.7
Sex	N (% non-missing)	485 (100.0)	861 (100.0)	0.0701	10.3	0.5
	Male, n (%)	316 (65.2)	518 (60.2)			
Smoking status	N (% non-missing)	483 (99.6)	850 (98.7)	0.1187	0.9	0.1
	Never, n (%)	42 (8.7)	93 (10.9)			
	Current, n (%)	186 (38.5)	284 (33.4)			
	Ex-smoker, n (%)	255 (52.8)	473 (55.6)			
BMI (kg/m^2)	N (% non-missing)	481 (99.2)	833 (96.7)	0.9473	1.7	0.6
	<18.5, n (%)	4 (0.8)	7 (0.8)			
	18.5 – <25, n (%)	73 (15.2)	125 (15.0)			
	25 – <30, n (%)	162 (33.7)	294 (35.3)			
	≥30, n (%)	242 (50.3)	407 (48.9)			
HbA1c (%), last value	N (% non-missing)	482 (99.4)	845 (98.1)	0.8028	0.8	0.3
	Mean (SD)	6.9 (1.1)	6.9 (1.2)			
	Median (IQR)	6.7 (1.1)	6.7 (1.1)			
Age (years) at DM diagnosis	N (% non-missing)	477 (98.4)	844 (98.0)	0.2032	6.9	0.2
	Mean (SD)	65.2 (9.5)	65.9 (9.8)			
	Median (IQR)	66.0 (13.0)	67.0 (14.0)			
Years since DM diagnosis	N (% non-missing)	477 (98.4)	844 (98.0)	0.0778	12.7	0.7
	<2 year, n (%)	109 (22.9)	238 (28.2)			
	2–10 years, n (%)	304 (63.7)	512 (60.7)			
	>10 years, n (%)	64 (13.4)	94 (11.1)			
Years since COPD diagnosis	N (% non-missing)	485 (100.0)	861 (100.0)	<0.0001	27.9	0.4
	>10 years, n (%)	59 (12.2)	195 (22.6)			
Cardiovascular disease	N (% non-missing)	485 (100.0)	861 (100.0)	0.8483	1.1	0.0
	Yes, n (%)	261 (53.8)	468 (54.4)			
Ischemic heart disease	N (% non-missing)	485 (100.0)	861 (100.0)	0.5069	3.8	0.0
	Yes, n (%)	167 (34.4)	312 (36.2)			
Hypertension	N (% non-missing)	485 (100.0)	861 (100.0)	0.1530	8.1	0.5
	Yes, n (%)	328 (67.6)	549 (63.8)			
Malignant cancer	N (% non-missing)	485 (100.0)	861 (100.0)	0.0019	17.3	0.4
	Yes, n (%)	109 (22.5)	135 (15.7)			
Charlson Comorbidity Index	N (% non-missing)	485 (100.0)	861 (100.0)	0.4384	1.1	0.1
	0–1, n (%)	127 (26.2)	257 (29.8)			
	2–5, n (%)	263 (54.2)	425 (49.4)			
	6–10, n (%)	49 (10.1)	84 (9.8)			
	11–20, n (%)	44 (9.1)	92 (10.7)			
	21–30, n (%)	2 (0.4)	3 (0.3)			

Supplementary Table 2. Baseline characteristics of diabetes progression cohort: unmatched

**Variable**		**LABD (n = 485)**	**ICS (n = 861)**	**P value***	**SMD (%)†**	**RCC (%)†**
	31–50, n (%)	0 (0.0)	0 (0.0)			
Nasal corticosteroids	≥1 prescription	39 (8.0)	88 (10.2)	0.1631	9.7	1.1
Antibiotic prescriptions	N (% non-missing)	485 (100.0)	861 (100.0)	0.0054	19.6	0.9
	0, n (%)	307 (63.3)	487 (56.6)			
	1, n (%)	108 (22.3)	193 (22.4)			
	2, n (%)	47 (9.7)	98 (11.4)			
	≥3, n (%)	23 (4.7)	83 (9.6)			
Acute OCS prescriptions	N (% non-missing)	485 (100.0)	861 (100.0)	0.0088	17.6	0.2
	0, n (%)	410 (84.5)	679 (78.9)			
	1, n (%)	57 (11.8)	117 (13.6)			
	≥2, n (%)	18 (3.7)	65 (7.5)			
OCS maintenance prescriptions‡	N (% non-missing)	485 (100.0)	861 (100.0)	0.1192	9.5	0.3
	Yes, n (%)	2 (0.4)	11 (1.3)			
All OCS prescriptions	N (% non-missing)	485 (100.0)	861 (100.0)	0.0120	19.2	0.0
	0, n (%)	408 (84.1)	674 (78.3)			
	1, n (%)	58 (12.0)	116 (13.5)			
	2, n (%)	13 (2.7)	42 (4.9)			
	≥3, n (%)	6 (1.2)	29 (3.4)			
SABA, mean daily dose (salbutamol equivalents), µg/day	N (% non-missing)	485 (100.0)	861 (100.0)	0.8116	4.9	0.3
	0, n (%)	215 (44.3)	357 (41.5)			
	>0 – ≤200, n (%)	170 (35.1)	311 (36.1)			
	>200 – ≤400, n (%)	48 (9.9)	98 (11.4)			
	>400 – ≤600, n (%)	19 (3.9)	31 (3.6)			
	>600, n (%)	33 (6.8)	64 (7.4)			
SABA inhaler devices	N (% non-missing)	485 (100.0)	861 (100.0)	0.0002	20.6	0.6
	0, n (%)	346 (71.3)	505 (58.7)			
	1, n (%)	45 (9.3)	116 (13.5)			
	2, n (%)	24 (4.9)	74 (8.6)			
	3, n (%)	10 (2.1)	28 (3.3)			
	≥4, n (%)	60 (12.4)	138 (16.0)			
SAMA prescriptions	N (% non-missing)	485 (100.0)	861 (100.0)	0.2277	7.2	0.9
	0, n (%)	368 (75.9)	672 (78.0)			
	1, n (%)	25 (5.2)	53 (6.2)			
	2, n (%)	47 (9.7)	63 (7.3)			
	3, n (%)	23 (4.7)	48 (5.6)			
	≥4, n (%)	22 (4.5)	25 (2.9)			
Glucose regulating medication prescribed	N (% non-missing)	485 (100.0)	861 (100.0)	0.9053	0.7	0.4
	Yes, n (%)	326 (67.2)	576 (66.9)			
Insulin prescribed	N (% non-missing)	485 (100.0)	861 (100.0)	0.1898	7.6	0.4
	Yes, n (%)	26 (5.4)	62 (7.2)			
Glucose regulating medication-prescriptions	N (% non-missing)	485 (100.0)	861 (100.0)	0.8517	1.0	0.7
	0, n (%)	174 (35.9)	307 (35.7)			
	1, n (%)	13 (2.7)	19 (2.2)			
	≥2, n (%)	298 (61.4)	535 (62.1)			
FEV_1 %predicted	N (% non-missing)	340 (70.1)	413 (48.0)	0.3471	9.8	1.1

Supplementary Table 2. Baseline characteristics of diabetes progression cohort: unmatched

Variable		LABD (n = 485)	ICS (n = 861)	P value*	SMD (%)†	RCC (%)†
	<30, n (%)	9 (2.6)	14 (3.4)			
	30–49%, n (%)	64 (18.8)	98 (23.7)			
	50–79%, n (%)	213 (62.6)	237 (57.4)			
	≥80%, n (%)	54 (15.9)	64 (15.5)			
Moderate-severe exacerbations	N (% non-missing)	485 (100.0)	861 (100.0)	0.0002	23.7	0.6
	0, n (%)	284 (58.6)	414 (48.1)			
	1, n (%)	122 (25.2)	236 (27.4)			
	≥2, n (%)	79 (16.3)	211 (24.5)			
ED respiratory attendances	N (% non-missing)	485 (100.0)	861 (100.0)	0.4474	4.4	0.1
	≥1, n (%)	4 (0.8)	11 (1.3)			
Inpatient respiratory admissions	N (% non-missing)	485 (100.0)	861 (100.0)	0.4198	4.7	0.1
	≥1, n (%)	12 (2.5)	28 (3.3)			
Outpatient respiratory visits	N (% non-missing)	485 (100.0)	861 (100.0)	0.0650	10.1	0.1
	≥1, n (%)	25 (5.2)	27 (3.1)			
MRC score	N (% non-missing)	460 (94.8)	725 (84.2)	0.0789	3.5	1.2
	1, n (%)	54 (11.7)	90 (12.4)			
	2, n (%)	196 (42.6)	316 (43.6)			
	3, n (%)	146 (31.7)	183 (25.2)			
	4, n (%)	54 (11.7)	114 (15.7)			
	5, n (%)	10 (2.2)	22 (3.0)			
GOLD risk group	N (% non-missing)	460 (94.8)	725 (84.2)	0.0281	13.7	1.7
	GOLD A	178 (38.7)	251 (34.6)			
	GOLD B	137 (29.8)	185 (25.5)			
	GOLD C	72 (15.7)	155 (21.4)			
	GOLD D	73 (15.9)	134 (18.5)			

*P values shown using Kruskal-Wallis equality-of-populations rank test or Pearson's χ^2 test of independent categories for continuous and categorical variables, respectively.

†An SMD ≤10% indicates sufficient balance between groups. The baseline variables with RCC ≥2%, which we defined as indicating bias potential, were selected for the direct matching attempts.

‡Patients prescribed maintenance OCS received 4 or fewer prescriptions in any given year, as per study eligibility criteria.

DM, diabetes mellitus; ED, emergency department; FEV_1, forced expiratory volume in 1 second; GOLD, Global Initiative for Chronic Obstructive Lung Disease; ICS, inhaled corticosteroid; IQR, interquartile range; LABD, long-acting bronchodilator; MRC, Medical Research Council dyspnea scale; OCS, oral corticosteroid; RCC, relative change in coefficient; SABA, short-acting β-agonist; SAMA, short-acting muscarinic antagonist; SMD, standardized mean difference; yr, during the baseline year.

Supplementary Table 3. Baseline characteristics of osteoporosis onset cohort: unmatched

Variable		LABD (n = 9334)	ICS (n = 26,820)	P value*	SMD (%)†	RCC (%)†
Index year	N (% non-missing)	9334 (100.0)	26,820 (100.0)	<0.0001	109.6	1.0
	Mean (SD)	2008.3 (3.7)	2002.9 (5.9)			
	Median (IQR)	2009.0 (5.0)	2004.0 (9.0)			
Age (years)	N (% non-missing)	9334 (100.0)	26,820 (100.0)	<0.0001	6.7	1.5
	≥40 – <60, n (%)	1731 (18.5)	5725 (21.3)			
	≥60 – <80, n (%)	6338 (67.9)	17,735 (66.1)			
	≥80, n (%)	1265 (13.6)	3360 (12.5)			
Age (years)	Mean (SD)	68.4 (9.9)	67.8 (10.2)	<0.0001	5.8	2.0
Sex	N (% non-missing)	9334 (100.0)	26,820 (100.0)	0.0029	3.6	2.7
	Male, n (%)	5664 (60.7)	15,803 (58.9)			
Smoking status	N (% non-missing)	9182 (98.4)	24,870 (92.7)	<0.0001	7.2	0.9
	Never, n (%)	562 (6.1)	2177 (8.8)			
	Current, n (%)	4095 (44.6)	10,893 (43.8)			
	Ex-smoker, n (%)	4525 (49.3)	11,800 (47.4)			
BMI (kg/m^2)	N (% non-missing)	8912 (95.5)	24,037 (89.6)	<0.0001	11.2	1.9
	<18.5, n (%)	334 (3.7)	1204 (5.0)			
	18.5 – <25, n (%)	3032 (34.0)	8893 (37.0)			
	25 – <30, n (%)	3029 (34.0)	8164 (34.0)			
	≥30, n (%)	2517 (28.2)	5776 (24.0)			
Years since COPD diagnosis	N (% non-missing)	9334 (100.0)	26,820 (100.0)	<0.0001	62.3	3.5
	>10 years, n (%)	1024 (11.0)	9713 (36.2)			
Diabetes	N (% non-missing)	9334 (100.0)	26,820 (100.0)	<0.0001	12.5	0.2
	Yes, n (%)	951 (10.2)	1801 (6.7)			
Cardiovascular disease	N (% non-missing)	9334 (100.0)	26,820 (100.0)	<0.0001	9.5	1.4
	Yes, n (%)	3532 (37.8)	8931 (33.3)			
Ischemic heart disease	N (% non-missing)	9334 (100.0)	26,820 (100.0)	<0.0001	5.6	0.5
	Yes, n (%)	2030 (21.7)	5226 (19.5)			
Hypertension	N (% non-missing)	9334 (100.0)	26,820 (100.0)	<0.0001	18.2	0.8
	Yes, n (%)	3759 (40.3)	8464 (31.6)			
Malignant cancer	N (% non-missing)	9334 (100.0)	26,820 (100.0)	<0.0001	19.8	1.1
	Yes, n (%)	1945 (20.8)	3598 (13.4)			
Charlson Comorbidity Index	N (% non-missing)	9334 (100.0)	26,820 (100.0)	<0.0001	0.7	0.1
	0–1, n (%)	7345 (78.7)	20,819 (77.6)			
	2–5, n (%)	1094 (11.7)	3713 (13.8)			
	6–10, n (%)	522 (5.6)	1052 (3.9)			
	11–20, n (%)	334 (3.6)	1135 (4.2)			
	21–30, n (%)	38 (0.4)	98 (0.4)			
	31–50, n (%)	1 (0.0)	3 (0.0)			
Nasal corticosteroids	≥1 prescription	610 (6.5)	2555 (9.5)	<0.0001	11.3	0.3
Antibiotic prescriptions	N (% non-missing)	9334 (100.0)	26,820 (100.0)	<0.0001	3.1	0.2
	0, n (%)	5625 (60.3)	16,136 (60.2)			
	1, n (%)	2146 (23.0)	5839 (21.8)			
	2, n (%)	969 (10.4)	2748 (10.2)			
	≥3, n (%)	594 (6.4)	2097 (7.8)			
Acute OCS	N (% non-missing)	9334 (100.0)	26,820 (100.0)	<0.0001	14.2	0.5

Supplementary Table 3. Baseline characteristics of osteoporosis onset cohort: unmatched

**Variable**		**LABD (n = 9334)**	**ICS (n = 26,820)**	**P value***	**SMD (%)†**	**RCC (%)†**
prescriptions	0, n (%)	7640 (81.9)	20,263 (75.6)			
	1, n (%)	1189 (12.7)	4629 (17.3)			
	≥2, n (%)	505 (5.4)	1928 (7.2)			
OCS maintenance prescriptions‡	N (% non-missing)	9334 (100.0)	26,820 (100.0)	0.0003	4.6	0.0
	Yes, n (%)	51 (0.5)	253 (0.9)			
All OCS prescriptions	N (% non-missing)	9334 (100.0)	26,820 (100.0)	<0.0001	13.8	0.5
	0, n (%)	7604 (81.5)	20,133 (75.1)			
	1, n (%)	1192 (12.8)	4635 (17.3)			
	2, n (%)	357 (3.8)	1296 (4.8)			
	≥3, n (%)	181 (1.9)	756 (2.8)			
SABA, mean daily dose (salbutamol equivalents), μg/day	N (% non-missing)	9334 (100.0)	26,820 (100.0)	0.0032	2.8	0.1
	0, n (%)	4037 (43.3)	11,646 (43.4)			
	>0 – ≤200, n (%)	3336 (35.7)	9273 (34.6)			
	>200 – ≤400, n (%)	1013 (10.9)	2849 (10.6)			
	>400 – ≤600, n (%)	376 (4.0)	1106 (4.1)			
	>600, n (%)	572 (6.1)	1946 (7.3)			
SABA inhaler devices	N (% non-missing)	9334 (100.0)	26,820 (100.0)	<0.0001	12.5	0.0
	0, n (%)	6073 (65.1)	15,408 (57.4)			
	1, n (%)	1123 (12.0)	3888 (14.5)			
	2, n (%)	556 (6.0)	2143 (8.0)			
	3, n (%)	284 (3.0)	946 (3.5)			
	≥4, n (%)	1298 (13.9)	4435 (16.5)			
SAMA prescriptions	N (% non-missing)	9334 (100.0)	26,820 (100.0)	<0.0001	8.9	0.4
	0, n (%)	7178 (76.9)	21,315 (79.5)			
	1, n (%)	539 (5.8)	1719 (6.4)			
	2, n (%)	674 (7.2)	1780 (6.6)			
	3, n (%)	645 (6.9)	1385 (5.2)			
	≥4, n (%)	298 (3.2)	621 (2.3)			
FEV_1 %predicted	N (% non-missing)	5727 (61.4)	8419 (31.4)	<0.0001	13.3	0.6
	<30, n (%)	204 (3.6)	441 (5.2)			
	30–49%, n (%)	1213 (21.2)	2241 (26.6)			
	50–79%, n (%)	3445 (60.2)	4537 (53.9)			
	≥80%, n (%)	865 (15.1)	1200 (14.3)			
Moderate-severe exacerbations	N (% non-missing)	9334 (100.0)	26,820 (100.0)	<0.0001	8.9	0.2
	0, n (%)	4968 (53.2)	13,293 (49.6)			
	1, n (%)	2585 (27.7)	7506 (28.0)			
	≥2, n (%)	1781 (19.1)	6021 (22.4)			
ED respiratory attendances	N (% non-missing)	9334 (100.0)	26,820 (100.0)	0.5776	0.7	0.0
	≥1, n (%)	107 (1.1)	327 (1.2)			
Inpatient respiratory admissions	N (% non-missing)	9334 (100.0)	26,820 (100.0)	0.3240	1.2	0.0
	≥1, n (%)	204 (2.2)	634 (2.4)			
Outpatient respiratory visits	N (% non-missing)	9334 (100.0)	26,820 (100.0)	<0.0001	11.4	0.3
	≥1, n (%)	443 (4.7)	700 (2.6)			
MRC score	N (% non-missing)	8404 (90.0)	16,730 (62.4)	<0.0001	2.0	0.1
	1, n (%)	1155 (13.7)	2133 (12.7)			

Supplementary Table 3. Baseline characteristics of osteoporosis onset cohort: unmatched

Variable		LABD (n = 9334)	ICS (n = 26,820)	P value*	SMD (%)†	RCC (%)†
	2, n (%)	4012 (47.7)	8417 (50.3)			
	3, n (%)	2227 (26.5)	3886 (23.2)			
	4, n (%)	866 (10.3)	1878 (11.2)			
	5, n (%)	144 (1.7)	416 (2.5)			
GOLD risk group	N (% non-missing)	8404 (90.0)	16,730 (62.4)	<0.0001	7.0	0.7
	GOLD A	3518 (41.9)	6642 (39.7)			
	GOLD B	2106 (25.1)	3789 (22.6)			
	GOLD C	1649 (19.6)	3908 (23.4)			
	GOLD D	1131 (13.5)	2391 (14.3)			

*P values shown using Kruskal-Wallis equality-of-populations rank test or Pearson's χ^2 test of independent categories for continuous and categorical variables, respectively.

†An SMD ≤10% indicates sufficient balance between groups. The baseline variables with RCC ≥2%, which we defined as indicating bias potential, were selected for the direct matching attempts.

‡Patients prescribed maintenance OCS received 4 or fewer prescriptions in any given year, as per study eligibility criteria.

ED, emergency department; FEV_1, forced expiratory volume in 1 second; GOLD, Global Initiative for Chronic Obstructive Lung Disease; ICS, inhaled corticosteroid; IQR, interquartile range; LABD, long-acting bronchodilator; MRC, Medical Research Council dyspnea scale; OCS, oral corticosteroid; RCC, relative change in coefficient; SABA, short-acting β-agonist; SAMA, short-acting muscarinic antagonist; SMD, standardized mean difference; yr, during the baseline year.

Supplementary Table 4. Additional demographic and clinical characteristics of matched patients in the diabetes onset cohort during the baseline year

Variable		LABD (n = 6540)	ICS (n = 11,430)	P value*	SMD (%)†	RCC (%)†
Body mass index (kg/m²)	N (% non-missing)	6540 (100.0)	11,430 (100.0)	0.0229	2.9	0.0
	<18.5, n (%)	211 (3.2)	336 (2.9)			
	18.5–<25, n (%)	2586 (39.5)	4655 (40.7)			
	25–<30, n (%)	2249 (34.4)	4031 (35.3)			
	≥30, n (%)	1494 (22.8)	2408 (21.1)			
Ischemic heart disease	N (% non-missing)	6540 (100.0)	11,430 (100.0)	0.1429	2.3	0.0
	Yes, n (%)	1249 (19.1)	2082 (18.2)			
Hypertension	N (% non-missing)	6540 (100.0)	11,430 (100.0)	0.2588	1.7	0.6
	Yes, n (%)	2321 (35.5)	3961 (34.7)			
Cancer	N (% non-missing)	6540 (100.0)	11,430 (100.0)	0.0006	5.3	0.1
	Yes, n (%)	1123 (17.2)	1740 (15.2)			
Charlson comorbidity index	N (% non-missing)	6540 (100.0)	11,430 (100.0)	0.0776	2.1	0.2
	0–1, n (%)	5613 (85.8)	9892 (86.5)			
	2–5, n (%)	435 (6.7)	755 (6.6)			
	6–10, n (%)	314 (4.8)	464 (4.1)			
	11–20, n (%)	157 (2.4)	292 (2.6)			
	21–30, n (%)	19 (0.3)	27 (0.2)			
	31–50, n (%)	2 (0.0)	0 (0.0)			
Nasal corticosteroids	≥1 prescription	418 (6.4)	854 (7.5)	0.0239	4.2	0.0
Acute OCS prescriptions/yr	N (% non-missing)	6540 (100.0)	11,430 (100.0)	<0.0001	8.5	1.6
	0, n (%)	5303 (81.1)	8877 (77.7)			
	1, n (%)	879 (13.4)	1767 (15.5)			
	≥2, n (%)	358 (5.5)	786 (6.9)			
OCS maintenance prescription(s) ‡	N (% non-missing)	6540 (100.0)	11,430 (100.0)	0.8495	0.3	0.1
	Yes, n (%)	38 (0.6)	69 (0.6)			
SABA, mean daily dose (salbutamol equivalent), μg/day	N (% non-missing)	6540 (100.0)	11,430 (100.0)	0.5817	0.4	0.1
	0, n (%)	2732 (41.8)	4706 (41.2)			
	>0 – ≤200, n (%)	2404 (36.8)	4339 (38.0)			
	>200 – ≤400, n (%)	717 (11.0)	1226 (10.7)			
	>400 – ≤600, n (%)	281 (4.3)	464 (4.1)			
	>600, n (%)	406 (6.2)	695 (6.1)			
SAMA prescriptions/yr	N (% non-missing)	6540 (100.0)	11,430 (100.0)	0.0002	6.2	0.9
	0, n (%)	5022 (76.8)	9004 (78.8)			
	1, n (%)	412 (6.3)	737 (6.4)			
	2, n (%)	495 (7.6)	836 (7.3)			
	3, n (%)	430 (6.6)	581 (5.1)			
	≥4, n (%)	181 (2.8)	272 (2.4)			
ED respiratory attendances/yr	N (% non-missing)	6540 (100.0)	11,430 (100.0)	0.0070	4.3	0.0
	≥1, n (%)	69 (1.1)	176 (1.5)			
Inpatient respiratory admissions/yr	N (% non-missing)	6540 (100.0)	11,430 (100.0)	0.0088	4.1	0.4
	≥1, n (%)	117 (1.8)	272 (2.4)			
Outpatient respiratory visits/yr	N (% non-missing)	6540 (100.0)	11,430 (100.0)	0.3032	1.6	0.0
	≥1 n (%)	203 (3.1)	324 (2.8)			

*P values shown using Kruskal-Wallis equality-of-populations rank test or Pearson's χ^2 test of independent categories for continuous and categorical variables respectively.

†An SMD ≤10% indicates sufficient balance between groups. The baseline variables with RCC ≥2%, which we defined as indicating bias potential, were selected for the direct matching attempts.

‡Patients prescribed maintenance OCS received 4 or fewer prescriptions in any given year, as per study eligibility criteria.

ED emergency department; ICS inhaled corticosteroid; LABD long-acting bronchodilator; OCS oral corticosteroid; RCC relative change in coefficient; SABA short-acting β-agonist; SAMA short-acting muscarinic antagonist; SMD standardized mean difference; yr during the baseline year.

Supplementary Table 5. Additional demographic and clinical characteristics of matched patients in the diabetes progression cohort during the baseline year

Variable		LABD (n = 324)	ICS (n = 480)	P value*	SMD (%)†	RCC (%)†
Age (years) at DM diagnosis	N (% non-missing)	324 (100.0)	480 (100.0)	0.4771	3.8	0.1
	Mean (SD)	65.3 (9.0)	65.7 (8.6)			
	Median (IQR)	66.0 (13.0)	66.5 (12.0)			
Years since DM diagnosis	N (% non-missing)	324 (100.0)	480 (100.0)	0.6184	7.0	2.2
	< 2 year n (%)	83 (25.6)	121 (25.2)			
	2-10 years n (%)	199 (61.4)	307 (64.0)			
	>10 years n (%)	42 (13.0)	52 (10.8)			
Ischemic heart disease	N (% non-missing)	324 (100.0)	480 (100.0)	0.8737	1.1	0.9
	Yes n (%)	115 (35.5)	173 (36.0)			
Hypertension	N (% non-missing)	324 (100.0)	480 (100.0)	0.7855	2.0	0.0
	Yes n (%)	223 (68.8)	326 (67.9)			
Cancer	N (% non-missing)	324 (100.0)	480 (100.0)	0.7052	2.7	0.6
	Yes n (%)	58 (17.9)	91 (19.0)			
Charlson comorbidity index	N (% non-missing)	324 (100.0)	480 (100.0)	0.9956	3.1	0.4
	0–1 n (%)	92 (28.4)	138 (28.7)			
	2–5 n (%)	170 (52.5)	247 (51.5)			
	6–10 n (%)	28 (8.6)	44 (9.2)			
	11–20 n (%)	33 (10.2)	50 (10.4)			
	21–30 n (%)	1 (0.3)	1 (0.2)			
Nasal corticosteroids	≥1 prescription	27 (8.3)	42 (8.7)	0.9154	3.0	0.1
Acute OCS prescriptions/yr	N (% non-missing)	324 (100.0)	480 (100.0)	0.2406	12.4	0.2
	0 n (%)	276 (85.2)	390 (81.3)			
	1 n (%)	37 (11.4)	63 (13.1)			
	≥2 n (%)	11 (3.4)	27 (5.6)			
OCS maintenance prescriptions‡	N (% non-missing)	324 (100.0)	480 (100.0)	0.8054	1.8	0.0
	Yes n (%)	1 (0.3)	2 (0.4)			
SABA mean daily dose (salbutamol equivalents) μg/day	N (% non-missing)	324 (100.0)	480 (100.0)	0.9734	5.1	0.2
	0 n (%)	132 (40.7)	205 (42.7)			
	>0 – ≤200 n (%)	124 (38.3)	181 (37.7)			
	>200 – ≤400 n (%)	38 (11.7)	50 (10.4)			
	>400 – ≤600 n (%)	11 (3.4)	16 (3.3)			
	>600 n (%)	19 (5.9)	28 (5.8)			
SAMA prescriptions/yr	N (% non-missing)	324 (100.0)	480 (100.0)	0.2288	16.8	2.0
	0 n (%)	242 (74.7)	379 (79.0)			
	1 n (%)	18 (5.6)	30 (6.3)			
	2 n (%)	35 (10.8)	31 (6.5)			
	3 n (%)	18 (5.6)	28 (5.8)			
	≥4 n (%)	11 (3.4)	12 (2.5)			
Glucose regulating medication ≥1 prescriptions	N (% non-missing)	324 (100.0)	480 (100.0)	0.3176	7.2	1.3
	Yes n (%)	216 (66.7)	336 (70.0)			
Insulin ≥1 prescriptions	N (% non-missing)	324 (100.0)	480 (100.0)	0.4458	5.4	0.7
	Yes n (%)	21 (6.5)	25 (5.2)			
Glucose regulating	N (% non-missing)	324 (100.0)	480 (100.0)	0.4440	9.2	1.3

medication number of prescriptions	0 n (%)	119 (36.7)	156 (32.5)			
	1 n (%)	6 (1.9)	11 (2.3)			
	≥2 n (%)	199 (61.4)	313 (65.2)			
ED respiratory attendances	N (% non-missing)	324 (100.0)	480 (100.0)	0.2662	8.3	0.0
	≥1 n (%)	2 (0.6)	7 (1.5)			
Inpatient respiratory admissions	N (% non-missing)	324 (100.0)	480 (100.0)	0.6642	3.1	0.1
	≥1 n (%)	9 (2.8)	11 (2.3)			
Outpatient respiratory visits	N (% non-missing)	324 (100.0)	480 (100.0)	0.6171	3.6	0.2
	≥1 n (%)	10 (3.1)	12 (2.5)			

*P values shown using Kruskal-Wallis equality-of-populations rank test or Pearson's χ^2 test of independent categories for continuous and categorical variables respectively.

†An SMD ≤10% indicates sufficient balance between groups. The baseline variables with RCC ≥2%, which we defined as indicating bias potential, were selected for the direct matching attempts.

‡Patients prescribed maintenance OCS received 4 or fewer prescriptions in any given year, as per study eligibility criteria.

DM diabetes mellitus; ED emergency department; ICS inhaled corticosteroid; LABD long-acting bronchodilator; LTRA leukotriene receptor antagonist; OCS oral corticosteroid; RCC relative change in coefficient; SABA short-acting β-agonist; SAMA short-acting muscarinic antagonist; SMD standardized mean difference; yr during the baseline year.

Supplementary Table 6. Additional demographic and clinical characteristics of matched patients in the osteoporosis onset cohort during the baseline year

Variable		LABD (n = 7279)	ICS (n = 12,619)	P value*	SMD (%)†	RCC (%)†
BMI (kg/m²)	N (% non-missing)	7279 (100.0)	12,619 (100.0)	0.0048	3.8	0.0
	<18.5 n (%)	199 (2.7)	310 (2.5)			
	18.5-<25 n (%)	2538 (34.9)	4622 (36.6)			
	25-<30 n (%)	2515 (34.5)	4428 (35.1)			
	≥30 n (%)	2027 (27.9)	3259 (25.8)			
Diabetes	N (% non-missing)	7279 (100.0)	12,619 (100.0)	0.9739	0.0	0.0
	Yes n (%)	678 (9.3)	1177 (9.3)			
Ischemic heart disease	N (% non-missing)	7279 (100.0)	12,619 (100.0)	0.0433	2.9	0.5
	Yes n (%)	1566 (21.5)	2564 (20.3)			
Hypertension	N (% non-missing)	7279 (100.0)	12,619 (100.0)	0.1861	1.9	0.8
	Yes n (%)	2833 (38.9)	4793 (38.0)			
Cancer	N (% non-missing)	7279 (100.0)	12,619 (100.0)	<0.0001	6.4	0.1
	Yes n (%)	1290 (17.7)	1936 (15.3)			
Charlson comorbidity index	N (% non-missing)	7279 (100.0)	12,619 (100.0)	0.3814	1.4	0.5
	0–1 n (%)	5829 (80.1)	10,190 (80.8)			
	2–5 n (%)	820 (11.3)	1382 (11.0)			
	6–10 n (%)	380 (5.2)	602 (4.8)			
	11–20 n (%)	226 (3.1)	414 (3.3)			
	21–30 n (%)	24 (0.3)	31 (0.2)			
	31–50 n (%)	0 (0.0)	0 (0.0)			
Nasal corticosteroids	≥1 prescription	486 (6.7)	1005 (8.0)	0.0036	4.9	0.1
Acute OCS prescriptions/yr	N (% non-missing)	7279 (100.0)	12,619 (100.0)	<0.0001	8.2	0.3
	0 n (%)	5908 (81.2)	9824 (77.9)			
	1 n (%)	975 (13.4)	1943 (15.4)			
	≥2 n (%)	396 (5.4)	852 (6.8)			
OCS maintenance prescriptions‡	N (% non-missing)	7279 (100.0)	12,619 (100.0)	0.2372	1.8	0.1
	Yes n (%)	37 (0.5)	81 (0.6)			
SABA mean daily dose (salbutamol equivalents) μg/day	N (% non-missing)	7279 (100.0)	12,619 (100.0)	0.9224	0.0	0.2
	0 n (%)	3063 (42.1)	5266 (41.7)			
	>0 - ≤200 n (%)	2694 (37.0)	4720 (37.4)			
	>200 - ≤400 n (%)	779 (10.7)	1377 (10.9)			
	>400 - ≤600 n (%)	300 (4.1)	498 (3.9)			
	>600 n (%)	443 (6.1)	758 (6.0)			
SAMA prescriptions/yr	N (% non-missing)	7279 (100.0)	12,619 (100.0)	<0.0001	6.9	0.3
	0 n (%)	5568 (76.5)	9947 (78.8)			
	1 n (%)	461 (6.3)	805 (6.4)			
	2 n (%)	556 (7.6)	912 (7.2)			
	3 n (%)	494 (6.8)	659 (5.2)			
	4+ n (%)	200 (2.7)	296 (2.3)			
ED respiratory attendances	N (% non-missing)	7279 (100.0)	12,619 (100.0)	0.0081	4.0	0.4
	≥1 n (%)	76 (1.0)	188 (1.5)			
Inpatient respiratory admissions	N (% non-missing)	7279 (100.0)	12,619 (100.0)	0.0062	4.1	0.2
	≥1 n (%)	139 (1.9)	317 (2.5)			
Outpatient	N (% non-missing)	7279 (100.0)	12,619 (100.0)	0.0404	3.0	0.3

respiratory visits	≥1 n (%)	246 (3.4)	361 (2.9)

*P values shown using Kruskal-Wallis equality-of-populations rank test or Pearson's χ^2 test of independent categories for continuous and categorical variables respectively.

†An SMD ≤10% indicates sufficient balance between groups. The baseline variables with RCC ≥2%, which we defined as indicating bias potential, were selected for the direct matching attempts.

‡Patients prescribed maintenance OCS received 4 or fewer prescriptions in any given year, as per study eligibility criteria.

ED emergency department; ICS inhaled corticosteroid; LABD long-acting bronchodilator; LTRA leukotriene receptor antagonist; OCS oral corticosteroid; RCC relative change in coefficient; SABA short-acting β-agonist; SAMA short-acting muscarinic antagonist; SMD standardized mean difference; yr during the baseline year.

Supplementary Table 7. Diabetes onset, diabetes progression, and osteoporosis onset in matched patients prescribed inhaled corticosteroid or long-acting bronchodilator therapy

Exposure group	N	Follow-up Time			Incidence Rate	
		Pt-Yr	Mean	SD	No. events	Per 100 Pt-Yr (95% CI)
**Diabetes onset**						
ICS vs LABD						
LABD	6540	17,538	2.7	2.0	184	1.05 (0.90-1.20)
ICS	11,430	51,704	4.5	3.1	647	1.25 (1.16-1.35)
ICS mean daily exposure						
<500 µg/day	9032	41,735	4.6	3.2	525	1.26 (1.15-1.36)
500–999 µg/day	2096	9122	4.4	3.1	111	1.22 (0.99-1.44)
≥1000 µg/day	298	817	2.7	2.0	11	1.35 (0.56-2.14)
ICS cumulative exposure						
Lowest tercile	6540	17,538	2.7	2.0	184	1.05 (0.90-1.20)
Middle tercile	5448	16,673	3.1	2.4	410	2.46 (2.22-2.69)
Highest tercile	5982	35,031	5.9	3.1	237	0.68 (0.59-0.76)
**Diabetes progression**						
ICS vs LABD						
LABD	324	500	1.5	1.6	186	37.20 (32.96-41.44)
ICS	480	905	1.9	2.1	301	33.26 (30.19-36.33)
ICS mean daily exposure						
<500 µg/day	381	761	2.0	2.2	240	31.54 (28.24-34.84)
500–999 µg/day	76	116	1.5	1.7	48	41.38 (32.42-50.34)
≥1000 µg/day	23	28	1.2	1.2	13	46.43 (27.96-64.90)
ICS cumulative exposure						
Lowest tercile	324	500	1.5	1.6	186	37.20 (32.96-41.44)
Middle tercile	213	167	0.8	1.2	175	104.80 (101.39-108.19)
Highest tercile	267	738	2.8	2.3	126	17.07 (14.36-19.79)
**Osteoporosis onset**						
ICS vs LABD						
LABD	7279	19,747	2.7	2.0	131	0.66 (0.55-0.78)
ICS	12,619	57,751	4.6	3.2	403	0.70 (0.63-0.77)
ICS mean daily exposure						
<500 µg/day	10,039	46,985	4.7	3.2	329	0.70 (0.62-0.78)
500–999 µg/day	2245	9853	4.4	3.1	67	0.68 (0.52-0.84)
≥1000 µg/day	332	889	2.7	2.0	7	0.79 (0.21-1.37)
ICS cumulative exposure						
Lowest tercile	7279	19,747	2.7	2.0	131	0.66 (0.55-0.78)
Middle tercile	5986	18,861	3.2	2.5	238	1.26 (1.10-1.42)
Highest tercile	6633	38,890	5.9	3.1	165	0.42 (0.36-0.49)

ICS inhaled corticosteroid; LABD long-acting bronchodilator; Pt-Yr patient-years.
ICS exposures are in fluticasone propionate dose-equivalents.

Supplementary Fig. 2. Hazard ratios (95% CIs) for cumulative inhaled corticosteroid (ICS) exposure (mg vs. reference value of 50 mg) among all patients for diabetes onset (N=11,430)

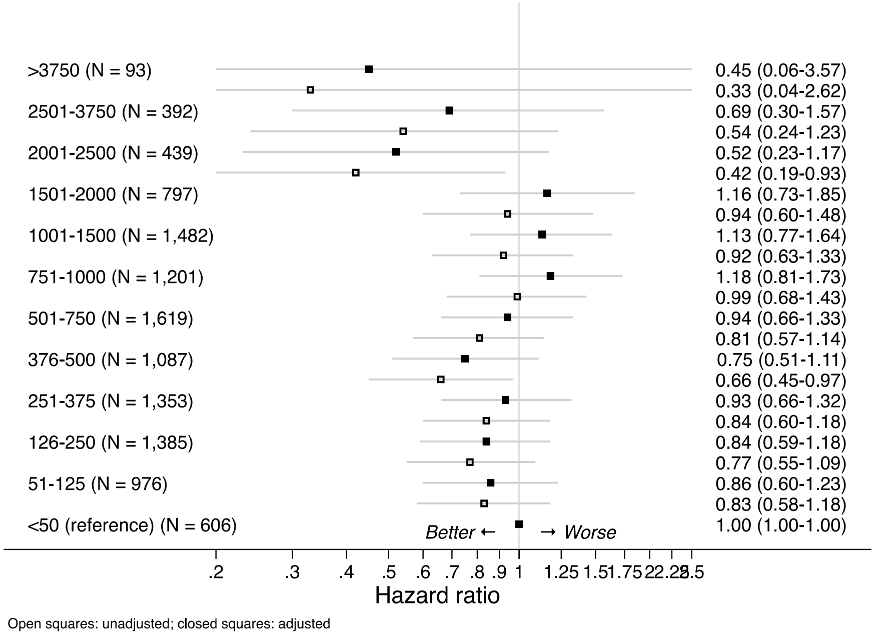

Supplementary Fig. 3. Hazard ratios (95% CIs) for cumulative inhaled corticosteroid (ICS) exposure (mg vs. reference value of 50 mg) among all patients for diabetes progression (N=480)

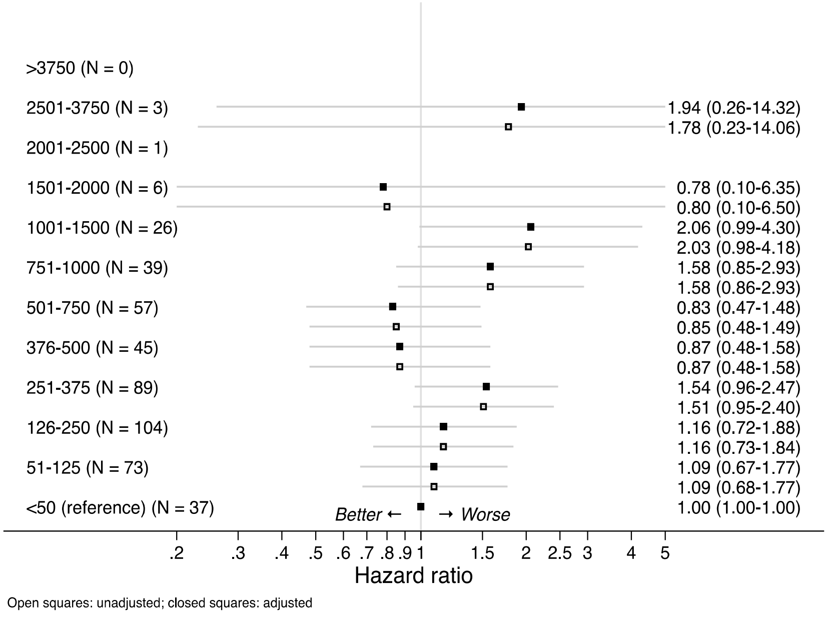

Supplementary Fig. 4. Hazard ratios (95% CIs) for cumulative inhaled corticosteroid (ICS) exposure (mg vs. reference value of 50 mg) among all patients for osteoporosis onset (N=12,619). (We note that the effect size for the highest exposure category could not be estimated.)

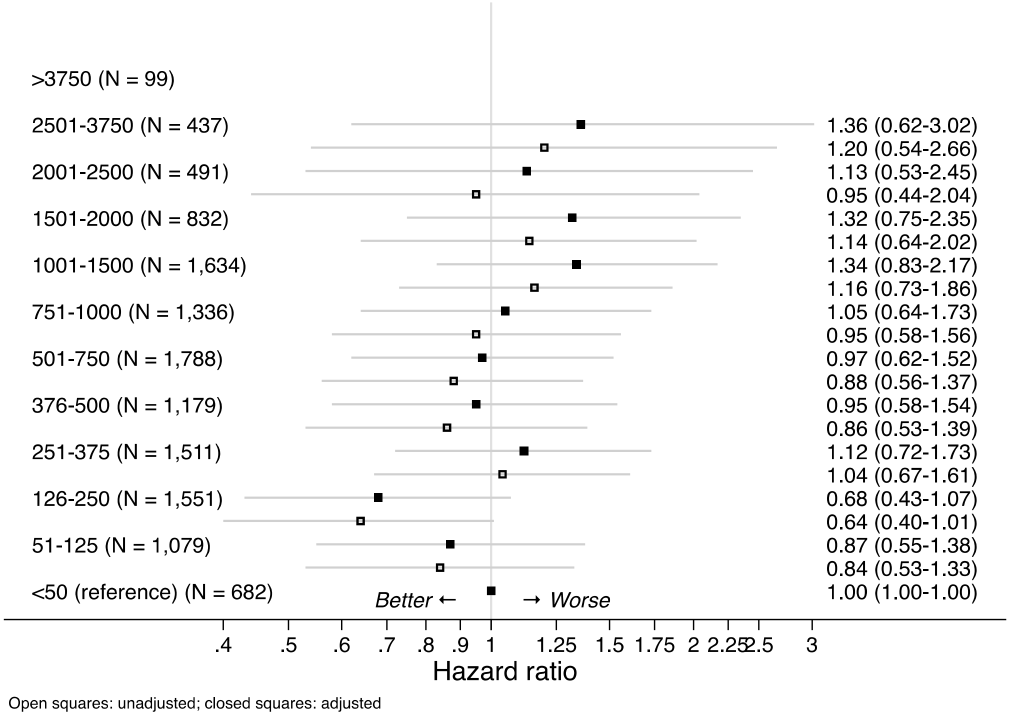

Supplement: Supplementary file 1 — Supplementary file [file 41533_2019_150_MOESM1_ESM.pdf]
